# Characterization of Resident Corneal Plasmacytoid Dendritic Cells and Their Pivotal Role in Herpes Simplex Keratitis

**DOI:** 10.1016/j.celrep.2020.108099

**Published:** 2020-09-01

**Authors:** Arsia Jamali, Kai Hu, Victor G. Sendra, Tomas Blanco, Maria J. Lopez, Gustavo Ortiz, Yureeda Qazi, Lixin Zheng, Aslihan Turhan, Deshea L. Harris, Pedram Hamrah

**Affiliations:** 1 Center for Translational Ocular Immunology, Tufts Medical Center, Tufts University School of Medicine, Boston, MA, USA; 2 Department of Ophthalmology, Tufts Medical Center, Tufts University School of Medicine, Boston, MA, USA; 3 Schepens Eye Research Institute, Department of Ophthalmology, Harvard Medical School, Boston, MA, USA; 4 Division of Immunology, Department of Microbiology and Immunobiology, Harvard Medical School, Boston, MA, USA; 5 Program in Immunology, School of Graduate Biomedical Sciences, Tufts University, Boston, MA, USA; 6 Cornea Service, Tufts New England Eye Center, Boston, MA, USA; 7 These authors contributed equally; 8 Present address: Department of Ophthalmology, Nanjing Drum Tower Hospital, the Affiliated Hospital of Nanjing University Medical School, Nanjing University, Nanjing, China; 9 Lead Contact

## Abstract

The presence and potential functions of resident plasmacytoid dendritic cells (pDCs) in peripheral tissues is unclear. We report that pDCs constitutively populate naïve corneas and are increased during sterile injuries or acute herpes simplex virus 1 (HSV-1) keratitis. Their local depletion leads to severe clinical disease, nerve loss, viral dissemination to the trigeminal ganglion and draining lymph nodes, and mortality, while their local adoptive transfer limits disease. pDCs are the main source of HSV-1-induced IFN-α in the corneal stroma through TLR9, and they prevent re-programming of regulatory T cells (Tregs) to effector ex-Tregs. Clinical signs of infection are observed in pDC-depleted corneas, but not in pDC-sufficient corneas, following low-dose HSV-1 inoculation, suggesting their critical role in corneal antiviral immunity. Our findings demonstrate a vital role for corneal pDCs in the control of local viral infections.

## In Brief

Jamali et al. show that the cornea, as an immune-privileged tissue, hosts resident pDCs, which mediate immunity against HSV-1 by secreting IFN-a via TLR9 and preserving Tregs. pDCs minimize the clinical severity of HSV-1 keratitis, infiltration of immune cells, nerve damage, and viral dissemination to TG and dLNs.

## Graphical Abstract

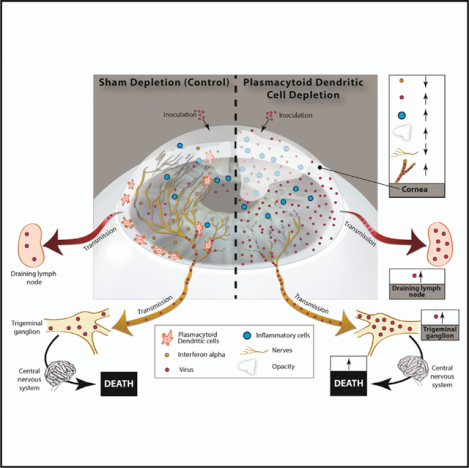

## INTRODUCTION

The cornea is among the very few tissues that enjoy immune privilege and can tolerate constant exposure to foreign antigens, allergens, and pathogens without eliciting significant immune responses during homeostasis. Although corneal immune privilege has historically been attributed to lack of resident immune cells during steady state, recent studies have demonstrated that the cornea is endowed with resident immune cells, including conventional dendritic cells (cDCs) and macrophages (Hamrah et al., 2002, 2003c; Brissette-Storkus et al., 2002).

Corneal infections can be associated with devastating consequences, among which herpes simplex virus 1 (HSV-1) keratitis is the leading cause of infectious blindness in developed countries (Liesegang, 2001). Interestingly, via unraveled mechanisms, clinical corneal manifestations of primary ocular HSV-1 infection are rare (Darougar et al., 1985; Liesegang et al., 1989). However, reactivation of latent virus in the trigeminal ganglion (TG) can result in corneal inflammation, ulceration, scarring, melting, perforation, and blindness (Liesegang, 1999; Rowe et al., 2013).

Constitutive expression of Toll-like receptor (TLR)7 and TLR9, along with interferon (IFN) response factor 7, enables pDCs to specialize in sensing microbial nucleic acids and uniquely equips them for contributing to defense against viral infections (Dalod et al., 2002; Honda et al., 2005; Ito et al., 2005; Smit et al., 2006), through production of high levels of type I IFNs (IFN-α/β) (Cella et al., 1999; Asselin-Paturel et al., 2001; Björck, 2001; Dzionek et al., 2001; Nakano et al., 2001). In mice, pDCs express PDCA-1, Siglec-H, CD45R/B220, Ly6C, Gr-1 (Ly6G/Ly6C), Ly49Q, and low to intermediate levels of CD11c and are negative for other lineage markers, such as CD19, CD3, and Ly6G (Asselin-Paturel et al., 2001; Nakano et al., 2001; Blasius et al., 2006; Zhang et al., 2006; Blasius et al., 2007; Caminschi et al., 2007; Segura et al., 2009; Reizis et al., 2011; Rogers et al., 2013). Human pDCs express CD123 (IL3R), BDCA-2, and BDCA-4 and lack CD11c (Dzionek et al., 2000, 2001). Although pDCs are confined mainly to the secondary lymphoid organs (McKenna et al., 2005), sparse numbers of pDCs can be found during steady state in non-lymphoid tissues (Lund et al., 2006; de Heer, 2004; Coates, 2004; Omatsu et al., 2005). Although pDCs are recognized as powerful orchestrators of innate and adaptive immune responses (Cella et al., 1999; Siegal et al., 1999; Cao and Liu, 2007; Villadangos and Young, 2008), their significance in priming effector or regulatory T cells (Tregs) in responses to viral pathogens remains controversial (Swiecki et al., 2010; Cervantes-Barragan et al., 2012; Lynch et al., 2018).

Herein, we show that human and murine corneas harbor a heretofore undetected population of tissue-resident pDCs during steady state and that their local depletion results in severe keratitis, poor viral clearance, increased inflammation, systemic viral dissemination, and mortality. Local adoptive transfer of pDCs enhances IFN-α levels, improves viral clearance in the cornea, and reduces severity of keratitis. Furthermore, we show that the impact of pDCs in HSV-1 keratitis can be attributed to a TLR9-dependent secretion of IFN-α and preservation of Tregs in the draining lymph nodes (dLNs).

## RESULTS

### The Cornea Is Endowed with Resident pDCs during Steady State

**Figure 1.**
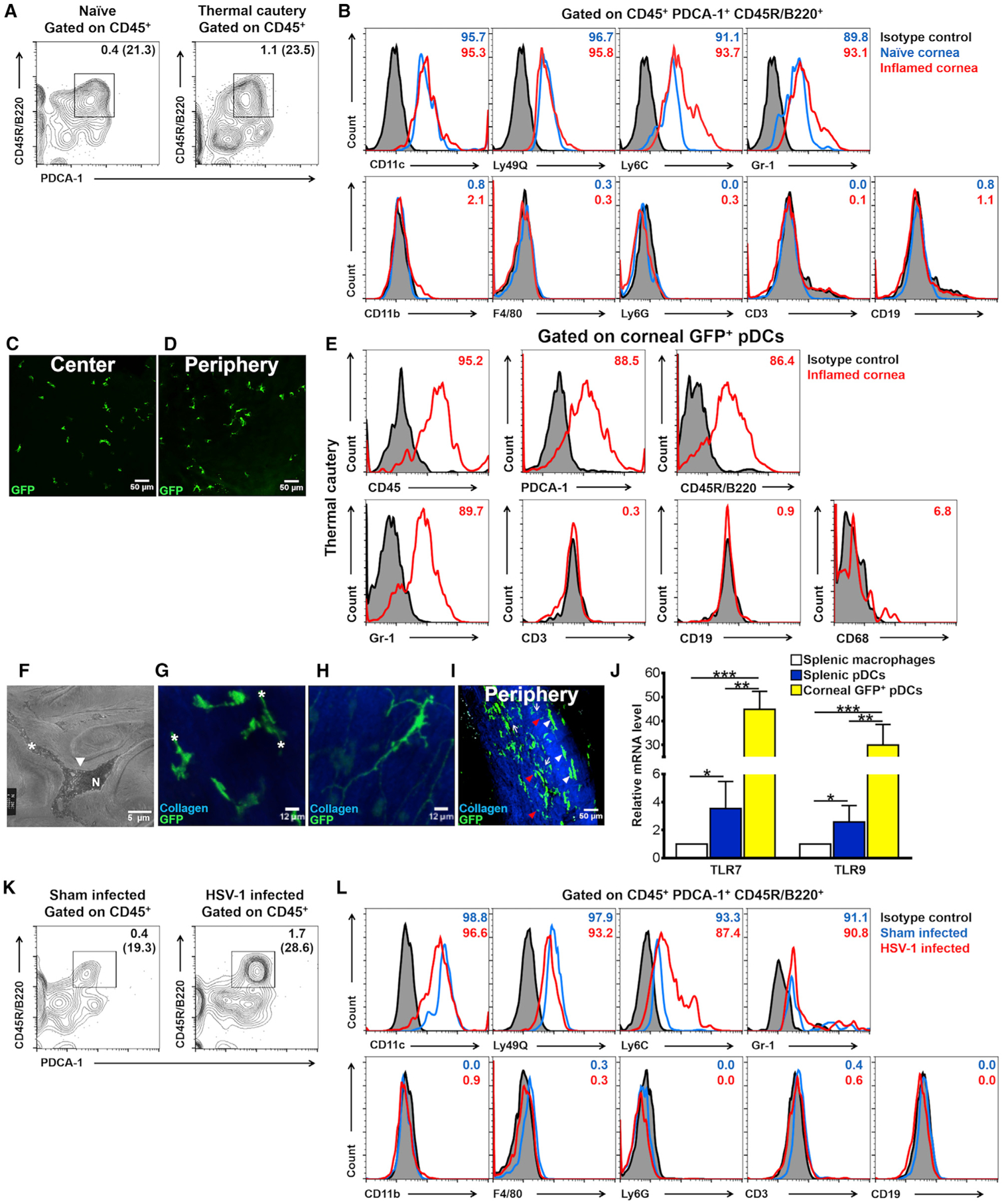
Presence of pDCs in Naïve Corneas and Alterations in Their Density upon Sterile Inflammation and Acute HSV-1 Keratitis (A and B) Flow cytometric analysis of pooled corneal single-cell suspensions of naïve mice (n = 15–20 corneas) and 3 days post-thermal cautery (n = 4–8 corneas). (A) Flow cytometry density plots illustrating PDCA-1^+^ CD45R/B220^+^ cells among CD45^+^ cells in naïve and inflamed corneas. Numbers represent the frequency of CD45^+^ PDCA-1^+^ CD45R/B220^+^ cells among total corneal single cells, and parentheses show the relative frequency of CD45^+^ PDCA-1^+^ CD45R/B220^+^ cells among CD45^+^ cells. (B) Flow cytometric histograms on the phenotype of CD45^+^ PDCA-1^+^ CD45R/B220^+^ cells in naïve and inflamed corneas. Depicted flow cytometry data are representative of three independent experiments. (C and D) Representative confocal micrograph of freshly excised unfixed corneas of DPE-GFP × RAG-1^−/−^ mice with GFP-tagged pDCs in the center (C) and periphery (D) of naïve corneas. Images are representative of n = 3 mice. (E) Flow cytometric histograms showing phenotype of GFP-tagged cells in the cornea of DPE-GFP × RAG-1^−/−^ mice on day 3 following thermal cautery. Depicted flow cytometry data are representative of three independent experiments. (F) TEM of a resident corneal pDC in a naïve WT C57BL/6 mouse (magnification 7,500x). White arrowhead, cell body; N, nucleus; white asterisk, cell processes extending from cell body. (G-I) MPM of freshly excised corneas. (G) Stub-like extensions from cell bodies (asterisks) depicted in naïve DPE-GFP × RAG-1^−/−^ mice. (H) A corneal cDC in a naïve CD11c-GFP-DTR mouse. (I) MPM of freshly excised inflamed cornea 3-days post-thermal cautery in DPE-GFP × RAG-1^−/−^ mice. Dendritiform processes (white arrows) and cell body (white arrowheads) are depicted. Another population of pDCs with round cell bodies without dendritiform processes (red arrowheads) is depicted as well. (J) Single-cell PCR on GFP-tagged pDCs from naïve corneas of DPE-GFP × RAG-1^−/−^ mice in comparison with naïve WT C57BL/6 splenic PDCA-1^+^ CD45R/B220^+^ Siglec-H^+^ pDCs and F4/80^+^ macrophages for mRNA levels of TLR7 and 9. Data represent three independent experiments. (K and L). Flow cytometric analysis of single-cell suspension of corneas of sham-infected (n = 15–20 pooled corneas) and HSV-1-infected (n = 4–8 pooled corneas) mice. (K) Density plots showing PDCA-1^+^ CD45R/B220^+^ cells among CD45^+^ cells. Numbers represent the frequency of CD45^+^ PDCA-1^+^ CD45R/B220^+^ cells among total corneal single cells, and parenthesis demonstrate relative frequency of CD45^+^ PDCA-1^+^ CD45R/B220^+^ cells among CD45^+^ cells. (L) Flow cytometric histograms on the phenotype of CD45^+^ PDCA-1^+^ CD45R/B220^+^ cells. Depicted flow cytometry data are representative of three independent experiments. Scale bars: 50 μm (C, D, and I), 12 μm (G and H), and 5 μm (F). pDC, plasmacytoid dendritic cell; N, cell nucleus; G, Golgi apparatus; white asterisk, thick process; white arrowhead, elongated cell body; red arrowhead, round cell body; white arrow, thin dendritiform process. Bars denote SD. *p < 0.05, **p < 0.01, and ***p < 0.001.

Recent work has identified a critical role for both TLR9 and type I IFNs in viral keratitis. However, resident corneal immune cells (Hamrah et al., 2002, 2003b, 2003c; Brissette-Storkus et al., 2002), such as cDCs and macrophages, do not express TLR9 in the cornea, and their contribution to secretion of type I IFNs upon viral infections is limited (Noisakran and Carr, 2000; Wuest et al., 2006; Chintakuntlawar et al., 2010; Conrady et al., 2011), suggesting that other hitherto unidentified bone marrow (BM)-derived cells may contribute to corneal antiviral immunity. Given that pDCs express TLR9 and can secrete large amounts of type I IFNs, we asked whether pDCs reside in naïve corneas. Performing flow cytometry on collagenase-digested single-cell suspensions of naïve wild-type (WT) C57BL/6 corneas, we observed that after gating out debris (Figure S1A), dead cells (Figure S1B), and doublets (Figure S1C), followed by gating on CD45^+^ cells (Figure S1D), a prominent population of PDCA-1^+^ CD45R/B220^+^ cells was apparent (constituting 21.3% of CD45^+^ cells; Figure 1A). In addition, we observed PDCA-1^+^ CD45R/B220^neg^ cells among CD45^+^ cells, constituting 20.8% of PDCA-1^+^ cells. Fluorescence minus one isotype controls showed that CD45^+^ PDCA-1^+^ CD45R/B220^+^ cells also expressed CD11c, Ly49Q, Ly6C, and Gr-1, but were negative for CD11b, F4/80, Ly6G, CD3, and CD19 (Figure 1B), indicating the presence of bona fide pDCs in the steady state cornea.

### Sterile Corneal Inflammation Results in Increased Density of pDCs

To determine the effect of inflammation on pDC homing, we first induced acute corneal inflammation in WT C57BL/6 mice by thermal cautery and performed flow cytometric analysis on corneal single-cell suspensions. After excluding debris, dead cells, and doublets (Figures S1E–S1G) and gating on CD45^+^ cells (Figure S1H), we observed that the frequency of CD45^+^ PDCA-1^+^ CD45R/B220^+^ cells was increased from 0.4% to 1.1% of total corneal cells on day 3 after induction of inflammation (Figure 1A). CD45^+^ PDCA-1^+^ CD45R/B220^+^ cells also expressed CD11c, Ly49Q, Ly6C, and Gr-1, but did not express CD11b, F4/80, Ly6G, CD3, and CD19 (Figure 1B), corroborating their pDC identity. Furthermore, we noted an increased density of CD45^+^ PDCA-1^+^ CD45R/B220^neg^ cells. Considering the expression of PDCA-1 by other immune and non-hematopoietic cells, particularly during inflammation, these cells may constitute a variety of cells (Bierly et al., 2008; Vinay et al., 2010, 2012; Bao et al., 2011; Blasius et al., 2006).

### GFP-Tagged pDCs Reside in Corneas of Transgenic DPE-GFP × RAG-1^−/−^ Mice

Having established that pDCs inhabit naïve corneas, we next took advantage of transgenic DPE-GFP × RAG-1^−/−^ mice, in which pDCs selectively express GFP (Iparraguirre et al., 2008), in order to examine their morphology and precise micro-anatomical distribution. Examining freshly excised unfixed corneas of DPE-GFP × RAG-1^−/−^ mice, we observed that central (Figure 1C) and peripheral corneas (Figure 1D) harbored resident GFP^+^ pDCs. To confirm the identity of GFP^+^ cells as pDCs, we performed flow cytometry on single-cell suspensions on day 3 following thermal cautery (to boost pDC numbers for robust analysis with fewer animals). As expected, fluorescent minus one staining indicated that corneal GFP^+^ cells expressed CD45, PDCA-1, CD45R/B220, and Gr-1; however, they were negative for CD3, CD19, and CD68, consistent with a pDC identity (Figure 1E). Next, to assess if type of inflammation may alter the identity of the GFP^+^ corneal cells, we assessed DPE-GFP × RAG-1^−/−^ corneas (WT C57BL/6 mice as controls) on day 7 following suture placement (Figures S1I–S1K). GFP^+^ cells costained for CD45, PDCA-1, CD45R/B220, and Gr-1, but not for CD3 and CD19 (Figure S1K), resembling our observation after thermal cautery. Conversely, we observed that GFP^+^ cells constituted 76.1% of CD45^+^ PDCA-1^+^ CD45R/B220^+^ cells (Figure S1L). To further validate the identity of GFP^+^ cells, we assessed the expression of E2–2/Tcf4 (a specific transcriptional regulator of the pDC lineage; Cisse et al., 2008) and CD45R/B220 by single-cell quantitative real-time PCR on sorted GFP^+^ corneal pDCs in comparison with WT splenic pDCs and macrophages (Figures S1M–S1P). Corneal GFP^+^ cells expressed high levels of E2–2/Tcf4 and CD45R/B220, further confirming their identity as pDCs (Figures S1Q).

### Multi-photon Microscopy (MPM) and Transmission Electron Microscopy (TEM) Reveal the Morphology and Location of Corneal pDCs in the Anterior Stroma

Next, seeking to assess corneal pDC morphology, we demonstrated using TEM that in naïve corneas, pDCs exhibited foot-like processes extending from the cell body (Figure 1F; Figures S2A and S2B, asterisk). Furthermore, an eccentric nucleus (Figure 1F; Figures S2A and S2B, denoted “N”) with predominant heterochromatin, numerous discernible vacuole-like endosomes, lysosomes, or other vesicles was noticeable. In comparison, cDCs exhibit a small central cell body with long thin dendrites, a central nucleus with a large amount of euchromatin surrounded by smooth endoplasmic reticulum, and numerous vacuole-like endosomes (Hamrah et al., 2003c).

MPM of corneal explants from DPE-GFP × RAG-1^−/−^ mice allowed three-dimensional (3D) reconstruction of pDC morphology without potential artifacts due to tissue processing (Ward and Rehg, 2014; Franek et al., 2016). MPM revealed that corneal pDCs were restricted to the anterior stroma, in close proximity to the epithelium, and were not detected in the corneal epithelium or posterior stroma (Figure 1G; Video S1). GFP^+^ pDCs exhibited a round cell body, lacking dendritic processes, but possessed round-ended, stub-like, extensions from the cell body (Figure 1G, asterisk) similar to TEM images, compared with resident GFP^+^ cDCs in naïve corneas of a CD11c-GFP-DTR mouse (Figure 1H). However, on day 3 after thermal cautery, in addition to pDCs with a similar morphology (Figure 1I), but with more elongated cell bodies (Figure 1I, white arrowheads) and numerous thin dendritiform processes (Figure 1I, arrows), some pDCs showed a rounder cell body lacking the aforementioned round-ended, stub-like extensions (Figure 1I, red arrowheads). Moreover, during chronic inflammation induced by suture placement, pDCs were confined to the anterior stroma (Video S2) and, similar to thermal cautery, could be observed with two distinct morphologies.

### Corneal pDCs Express TLR7 and TLR9

pDCs are known for their unique pattern recognition receptor repertoire, TLR7 and TLR9. Considering general concerns regarding the accuracy of TLR immunohistochemistry, we performed single-cell quantitative real-time PCR on sorted corneal GFP-tagged pDCs from naïve DPE-GFP × RAG-1^−/−^ mice compared with sorted WT splenic pDCs and macrophages to corroborate their TLR7 and TLR9 expression (Figures S1M–S1P). mRNA expression for TLR7 and TLR9 by corneal pDCs was higher than for both macrophages and splenic pDCs (Figure 1J). As peripheral barrier tissue-resident pDCs may serve as the first line of defense against invading pathogens, corneal pDCs may have higher expression of TLRs that serve as sensors for microbial attacks.

### Distribution of pDCs in Naïve and Inflamed Corneas

Resident immune cell density, including that of cDCs and macrophages in the cornea, follows a decremental gradient toward the center (Hamrah et al., 2002, 2003c). Thus, in order to assess the differential tissue distribution of pDCs in WT corneas, we performed immunofluorescence staining followed by confocal microscopy on corneal whole mounts of WT C57BL/6 mice. Considering that flow cytometry analysis indicated that a significant fraction of corneal CD45^+^ PDCA-1^+^ CD45R/B220^+^ cells are in fact pDCs, quantification of CD45^+^ PDCA-1^+^ or CD45^+^ PDCA-1^+^ CD45R/B220^+^ cell density by confocal microscopy should closely mirror that of pDCs. In line with our findings in DPE-GFP × RAG-1^−/−^ mice, CD45^+^ PDCA-1^+^ cells were detected at a higher density in the corneal periphery (Figures S2C–S2E) than the center (Figures S2F–S2H). Triple staining with CD45, PDCA-1, and another pDC-associated marker, CD45R/B220 (Figure S2I), confirmed the presence of CD45^+^ cells, co-expressing PDCA-1 and CD45R/B220 (Nakano et al., 2001; Gilliet et al., 2002). Staining with isotype controls for PDCA-1 (Figure S2J) or CD45R/B220 (Figure S2K) was negative.

To assess how acute and chronic inflammation affect distribution of corneal CD45^+^ PDCA-1^+^ cells, we stained whole mounts with CD45 and PDCA-1 at different time points after induction of inflammation (Figures S2L–S2N). We also performed triple staining with CD45, PDCA-1, and CD45R/B220 at one time point after induction of inflammation to confirm that the majority of CD45^+^ PDCA-1^+^ cells also express CD45R/B220 (Figure S2O). We observed that after an initial decrease in the density of CD45^+^ PDCA-1^+^ cells in both corneal periphery and center at day 1, they increased in the periphery and central cornea following both cautery and suture placement (Figure S2P). In the chronic inflammation model, CD45^+^ PDCA-1^+^ cells continued to increase, whereas in the acute inflammation model, in which localized epithelial defects heal after a few days (Williamson et al., 1987), CD45^+^ PDCA-1^+^ cells peaked by day 3 and then returned to steady-state numbers by day 14 (Figure S2P).

### Corneal pDCs Density Increases during Acute HSV-1 Keratitis

Next, we aimed to assess the role of corneal pDCs during HSV keratitis. We initially evaluated the impact of acute HSV keratitis on pDC density using flow cytometry. We observed a dramatic increase in the frequency of CD45^+^ PDCA-1^+^ CD45R/B220^+^ cells at day 5 post-inoculation (p.i.) from 0.4% of corneal cells to 1.7% (Figure 1K). Consistent with our findings on naïve and inflamed corneas, CD45^+^ PDCA-1^+^ CD45R/B220^+^ cells expressed CD11c, Ly49Q, Ly6C, and Gr-1, but they were negative for CD11b, F4/80, Ly6G, CD3, and CD19 (Figure 1L).

To assess tissue distribution of pDCs during HSV-1 keratitis, we next performed confocal microscopy on corneal whole mounts of sham- and HSV-1-infected corneas at different time points (Figure S2Q). As early as day 1 p.i., PDCA-1^+^ cell density significantly increased in both peripheral and central corneas in comparison with sham-infected corneas, with a more prominent increase at later time points (Figure S2R).

### Corneal Inflammation Alters Migratory Kinetics of pDCs

**Figure 2.**
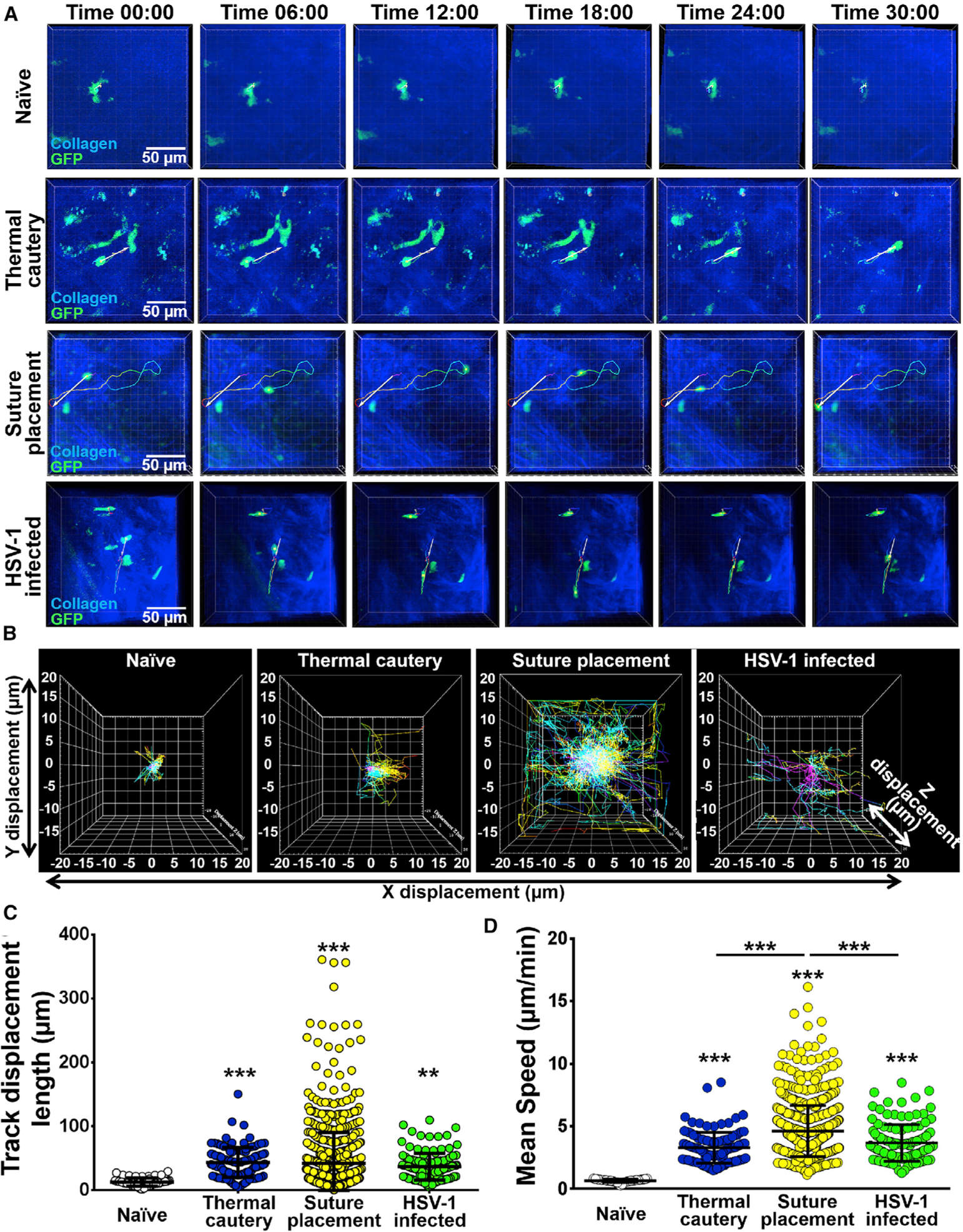
Assessment of the Kinetics of pDCs in Naïve Corneas and following Sterile Inflammation and HSV-1 Keratitis (A) Representative tracking of GFP-tagged pDCs generated by offline analysis of 4D intravital microscopy on naïve corneas, 3 days post-thermal cautery, 7 days following suture placement, and 5 days after 2 × 10^6^ PFU McKrae HSV-1-inoculated corneas of DPE-GFP × RAG-1^−/−^ mice (n = 3–5/condition). Scale bar: 50 μm. (B-D) Representative trajectories of individual pDCs following the alignment of their starting positions (B). Track displacement length (C) and mean speed (D) of pDCs in aforementioned conditions. Each dot represents one cell. Bars denote SD. **p < 0.01 and ***p < 0.001.

In order to assess if inflammation affects migratory kinetics of corneal pDCs, we performed intravital MPM on naïve DPE-GFP × RAG-1^−/−^ mice (Video S3), after induction of sterile inflammation by thermal cautery (Video S4) and suture placement (Video S5), as well as following HSV-1 inoculation (Video S6). Figure 2A demonstrates representative tracking of cell movement for pDCs in different conditions. Cell displacement for pDCs was increased after inflammation (Figure 2B). In fact, although only subtle movements were observed in pDCs in naïve corneas (Video S3), pDCs traveled longer distances in inflamed corneas (Videos S4, S5, and S6; Figure 2C).

Furthermore, corneal pDCs showed a higher mean speed during inflammation (Figure 2D; p < 0.001). Median speeds of pDCs after thermal cautery (3.2 μm/min) and HSV-1 infection (3.4 μm/min) were comparable, with slightly higher speed after suture placement (4.2 μm/min), but were lower than previously reported for cDCs in dLNs (Bousso and Robey, 2003; Mempel et al., 2004), likely because of high density of collagen fibers in the cornea that might hinder cell movements. Furthermore, we observed no significant difference in the meandering index (a measure for directionality) of corneal pDCs after thermal cautery, suture placement, and HSV-1 inoculation. These findings suggest that although resident corneal pDCs exhibit minimal movements during steady state, they are more motile in inflammatory microenvironments.

### Corneal pDC Depletion Exacerbates Severity of Acute HSV-1 Keratitis

In order to investigate the functional role of pDCs in acute HSV keratitis, we locally depleted corneal pDCs in BDCA2-DTR mice, in which pDCs are selectively ablated following exposure to diphtheria toxin (DT) (Swiecki et al., 2010). Administration of 30 ng DT did not alter the structural integrity of the cornea (Figure S3A). A single subconjunctival injection of DT led to approximately 97% depletion of corneal pDCs (Figures S3B and -S3C); however, pDCs gradually repopulated, reaching more than 80% of their baseline density by day 6 (Figure S3C). Therefore, we repeated DT injections every 2 days in order to continuously maintain local corneal pDC depletion (Figure S3C). Notably, pDC depletion remained confined to the cornea, as the density of pDCs in the BM, dLN, and TG remained unchanged (Figure S4).

**Figure 3.**
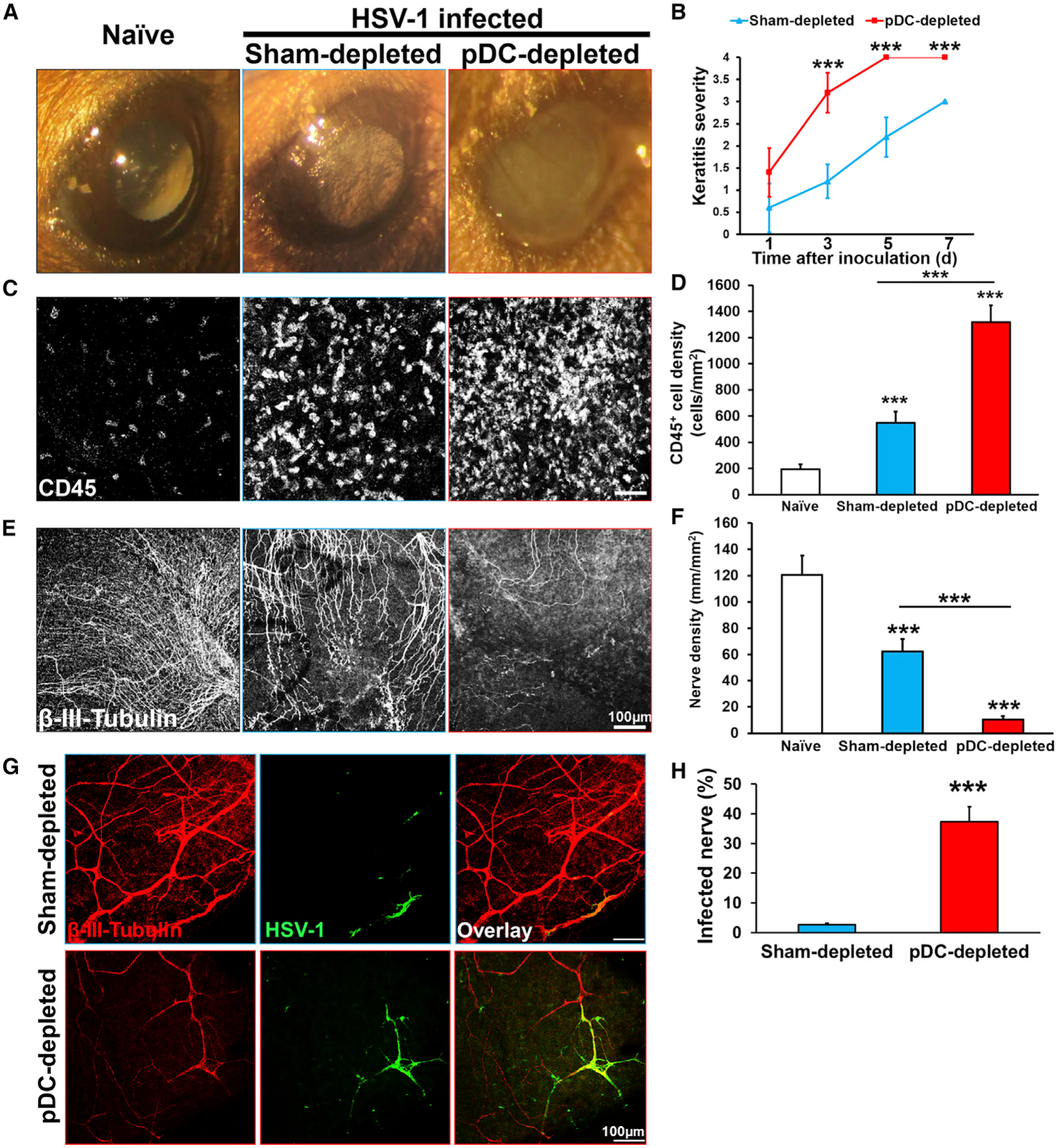
Local pDCs Depletion Is Associated with Severe HSV-1 Keratitis, Increased Corneal Damage, and Corneal Nerve Infection (A and B) Representative image of naïve cornea (left), sham-depleted HSV-1-infected (middle), and pDC-depleted HSV-1-infected (right) corneas on day 5 post-inoculation (A). Quantification of clinical keratitis severity (n = 5/time point; B). (C-F) Representative confocal micrographs of whole-mounted corneas stained with pan-leukocyte marker CD45 on day 5 post-inoculation (C) and neuronal marker β-III-tubulin on day 1 post-inoculation (E) in naïve cornea (left), sham-depleted HSV-1-infected (middle), and pDC-depleted HSV-1-infected (right). Quantification of confocal micrographs presenting the density of immune cells (D) and corneal nerves (F) (n = 5/time point). (G and H) Confocal micrograph of sham- and pDC-depleted corneas depicting co-localization of HSV-1 and corneal nerves on day 1 post-inoculation (G). Quantification of co-localized HSV-1 and corneal nerves at day 1 post-infection (n = 5/group; H). Scale bars: 100 μm (C, E, and G). Bars denote SD. ***p < 0.001.

To assess the role of corneal pDCs during acute HSV keratitis, pDC were depleted 2 days prior to ocular HSV-1 inoculation. Clinical keratitis severity, judged by the degree of corneal opacification, was markedly exacerbated in pDC-depleted mice as early as day 3 p.i. (Figures 3A and 3B). Confocal micrographs of HSV-infected corneal whole mounts showed increased CD45^+^ immune cell density in pDC-depleted corneas (Figures 3C and 3D).

Patients with HSV-1 keratitis present with a dramatic loss of subbasal corneal nerves within the first week of infection (Hamrah et al., 2010), a finding that has more recently been confirmed in murine models of HSV keratitis (Yun et al., 2014; Chucair-Elliott et al., 2015; Hu et al., 2015). In order to understand the potential role of pDCs in this process, HSV-infected corneas were stained for the neuronal marker β-III-tubulin, and corneal nerve density was assessed. As early as day 1 p.i., more profound nerve loss was observed in pDC-depleted corneas (Figures 3E and 3F) compared with controls. Next, we sought to pinpoint the cellular reservoir for HSV in pDC-depleted corneas by double staining HSV-1-inoculated corneas for HSV-1 (using a polyclonal antibody against all viral envelope glycoproteins and at least one core protein) and β-III-tubulin (Figure 3G). Despite the augmented nerve loss in HSV-infected pDC-depleted corneas, the proportion of corneal nerves that contained detectable HSV-1 was dramatically enhanced in pDC-depleted corneas on day 1 p.i. (Figure 3H). Thus, pDC depletion and subsequent higher viral titers result in increased nerve infection.

### Depletion of Corneal pDCs Results in Increased HSV-1 Spread and Mortality

**Figure 4.**
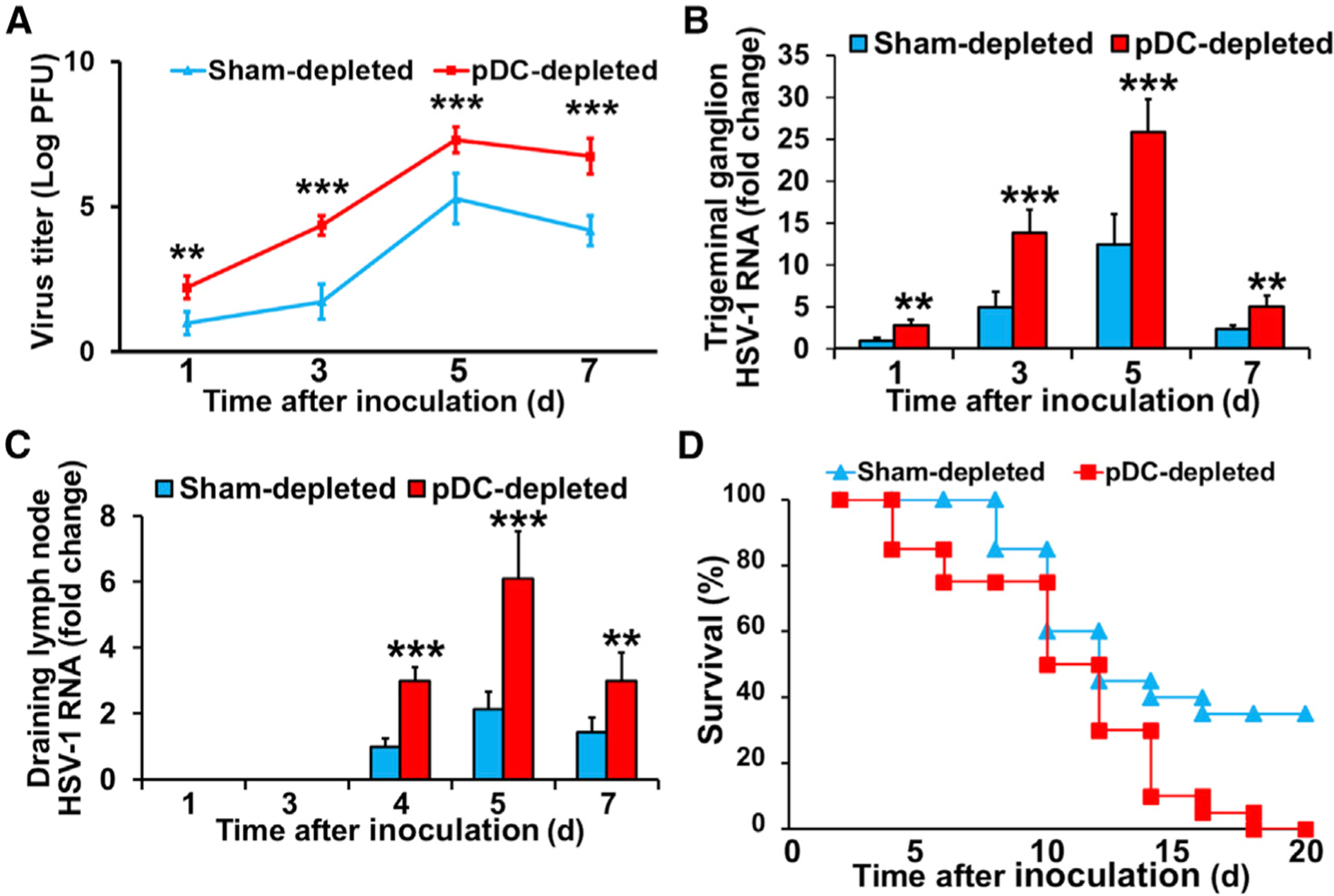
Local pDC Depletion Is Associated with Increased Local HSV-1 Load, Viral Transmission to the Trigeminal Ganglion and Draining Lymph Nodes, and Reduced Survival (A) Viral titers in corneal homogenates of sham- and pDC-depleted corneas (n = 5/time point). (B and C) HSV-1 gB RNA levels in TG (B) and dLN (C) of sham- and pDC-depleted mice (n = 6/time point). (D) Survival analysis of sham- and pDC-depleted mice following HSV-1 inoculation (n = 20/group). Bars denote SD. **p < 0.01 and ***p < 0.001.

In order to determine if the increased keratitis severity was associated with increased viral load, HSV titers were measured in homogenized HSV-inoculated corneas. Compared with the sham-depleted group, pDC depletion was associated with higher viral titers as early as day 1 p.i. and remained elevated through day 7 p.i. (Figure 4A). Next, we assessed whether the increased neuronal viral load in pDC-depleted corneas affected viral transmission to the TG and dLNs. Quantitative real-time PCR showed a 2.8-fold increase in HSV-1 gB RNA in the TG following corneal pDC depletion as early as day 1 p.i., peaking on day 5 p.i. Moreover, viral clearance from the TG was delayed as evidenced by greater HSV gB RNA level on day 7 (Figure 4B). Similarly, increased HSV RNA was detected in the dLNs at day 4 p.i., peaking on day 5, and decreasing on day 7, with pDC depletion resulting in significantly increased HSV gB RNA levels and delayed viral clearance (Figure 4C).

HSV-1 keratitis by the virulent McKrae strain of HSV-1 can result in viral transmission to the CNS and death (Knotts et al., 1974). Of note, we observed that pDC-depleted animals began to die on day 4 p.i., and none survived beyond day 18; however, approximately 35% of the control group were still alive on day 20 (Figure 4D). These findings highlight a critical protective role for corneal pDCs against local viral invasion, dissemination, and prevention of death.

### Corneal pDCs Secrete IFN-α in a TLR9-Dependent Fashion in Acute HSV-1 Keratitis

**Figure 5.**
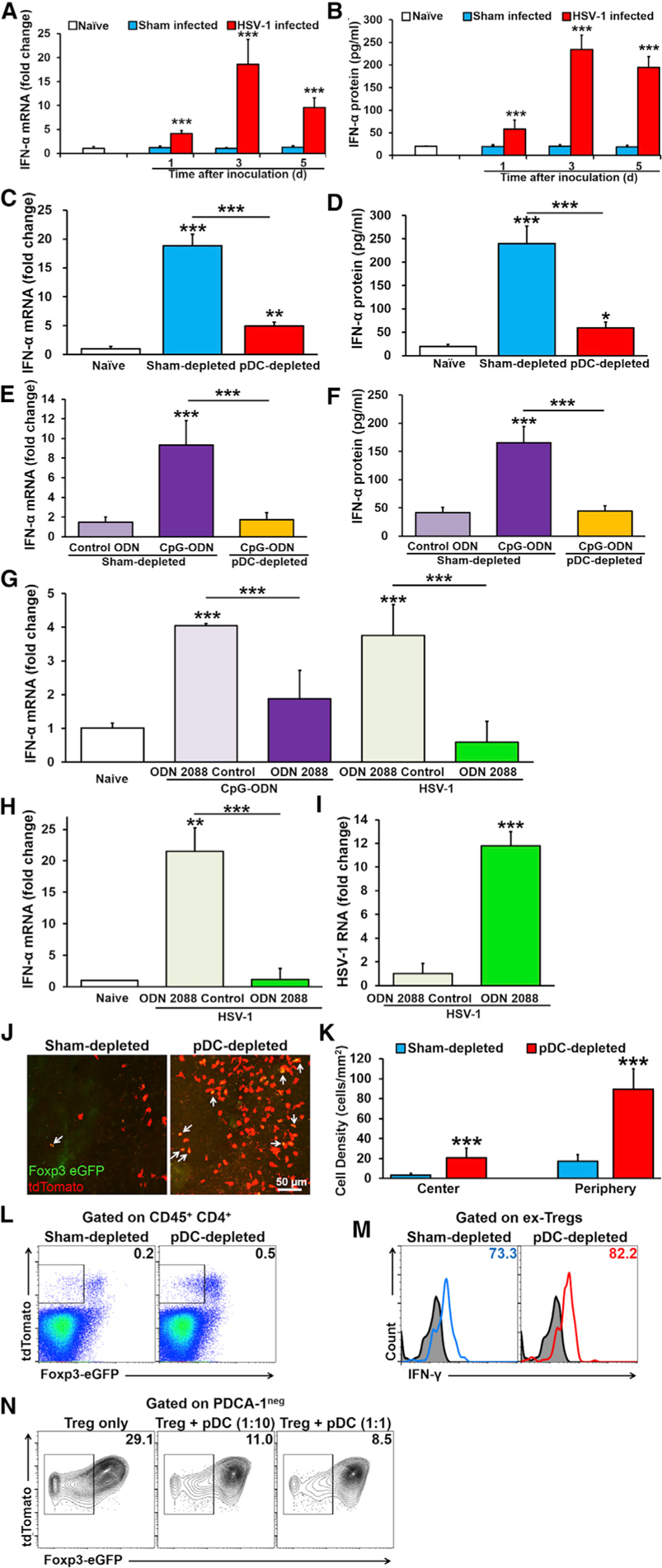
pDCs Secrete High Levels of IFN-α in the Cornea via TLR9 Signaling and Promote Treg Survival during Acute HSV-1 Keratitis (A and B) IFN-α mRNA (A) and protein (B) levels in whole corneal homogenates of naïve , sham-infected, and HSV-1-infected WT C57BL/6 mice (n = 5/time point). (C-F) IFN-α mRNA (C) and protein (D) levels on day 3 post-HSV-1 infection in whole corneas of naïve, sham-depleted, and pDC-depleted corneas (n = 5/group). IFN-α mRNA (E) and protein (F) levels in the corneal stroma of sham- and pDC-depleted corneas 24 h after inoculation with 20 μg control ODN or synthetic TLR9 agonist CpG-ODN (n = 5/group). (G) *In vitro* culture of splenic GFP^+^ pDCs obtained from DPE-GFP × RAG-1^−/−^ mouse 24 h after the following treatments: (1) 1 μg/mL control oligonucleotide 1826, (2) 1 μg/mL CpG-ODN (TLR9 agonist) and 10 μg/mL ODN 2088 control (TLR9 antagonist control), (3) 1 μg/mL CpG-ODN and 10 μg/mL ODN 2088 (TLR9 antagonist), (4) 10^5^ PFU UV-irradiated McKrae HSV-1 and 10 μg/mL ODN 2088 control, and (5) 10^5^ PFU UV-irradiated McKrae HSV-1 and 10 μg/mL ODN 2088. Data represents three independent experiments. (H and I) Relative mRNA levels of IFN-α (H) and gB (I) in the cornea on day 3following HSV-1 inoculation and subconjunctival administration of 10 μg ODN 2088 control (TLR9 antagonist control) or 10 μg ODN 2088 (TLR9 antagonist; n = 3 or 4/group). (J and K) Representative confocal micrographs of corneal whole mounts of BDCA-2-DTR and Treg FM chimeric mice presenting infiltration of both Foxp3-eGFP^+^ tdTomato^+^ Tregs (white arrows) and Foxp3-eGFP^ne9^ tdTomato^+^ ex-Tregs (J). Quantification of ex-Tregs in the corneas (n = 7 or 8/group) (K). (L and M) Representative flow cytometry dot plots of the dLNs of BDCA-2-DTR and Treg FM chimeric mice in pDC-depleted mice compared with control chimeric mice receiving subconjunctival PBS (L). Representative flow cytometric histograms indicating expression of IFN-γ by Foxp3-eGFP^neg^ tdTomato^+^ ex-Tregs in the dLNs of pDC-depleted and control chimeric mice (M). (N) *In vitro* co-culture of splenic Tregs and pDCs obtained from Treg FM and WT mice, respectively with 10^5^ PFU UV-irradiated McKrae HSV-1 and 1 ng/mLTGF-β1 for 3 days. Flow cytometry plots are representative of three independent experiments. Scale bar: 50 μm. Bars denote SD. *p < 0.05, **p < 0.01, and ***p< 0.001.

To explore the mechanism by which pDCs protect against HSV-1 in the cornea, we next assessed their IFN-α expression (Cella et al., 1999; Asselin-Paturel et al., 2001; Björck, 2001; Dzionek et al., 2001; Nakano et al., 2001; Dalod et al., 2002). Following ocular inoculation of HSV-1 in WT mice, both IFN-a mRNA (Figure 5A) and protein levels (Figure 5B) were increased on day 1 p.i. and peaked on day 3. As expected, pDC depletion was associated with a markedly attenuated IFN-a response to HSV infection on day 3, at both mRNA (Figure 5C) and protein levels (Figure 5D), suggesting that corneal IFN-α secretion during acute HSV keratitis is largely pDC dependent.

Next, to test whether IFN-α response to HSV-1 by pDCs was dependent on TLR9, an endosomal sensor of CpG-rich microbial DNA that is highly expressed in pDCs (Vremec et al., 2007), we treated sham- or pDC-depleted corneas with control oligonucleotide (ODN) 1826 or phosphorothioate CpG 1826 ODN (CpG-ODN; a synthetic TLR9 agonist) and measured IFN-α mRNA and protein levels in the corneal stroma 24 h after ODN inoculation. In sham-depleted corneas, treatment with CpG-ODN was associated with increased stromal IFN-α mRNA (Figure 5E) and protein levels (Figure 5F), indicating that as expected, TLR9 induces type I IFN secretion (Lund et al., 2003; Vremec et al., 2007). In contrast, in pDC-depleted corneas, the increase in IFN-α mRNA and protein levels following CpG-ODN treatment was attenuated, demonstrating a vital role of pDCs in IFN-α secretion in the corneal stroma following TLR9 stimulation.

Next, to assess if the pDC response to HSV-1 is TLR9 mediated, we exposed sorted splenic GFP^+^ pDCs (Figure S1R) to CpG-ODN or UV-inactivated HSV-1 in the presence of either TLR9 antagonist (ODN 2088) or ODN 2088 negative control (ODN 2088 control) (Yoshizaki et al., 2016). In line with our *in vivo* experiments, *in vitro* pDC exposure to either CpG-ODN or HSV-1 induced robust expression of IFN-α, which was effectively blocked by the TLR9 antagonist (Figure 5G), indicating that the pDCs response to HSV-1 requires direct TLR9 stimulation. Having observed the *in vitro* effects of TLR9 on pDC-derived IFN-α response, we measured IFN-α and viral gB levels in the cornea following HSV-1 infection following blockade of TLR9 through the TRL9 antagonist or its control. Although HSV-1 keratitis was accompanied by a considerable increase in corneal IFN-α levels, the increase was substantially inhibited by application of a TLR9 antagonist (Figure 5H). Furthermore, administration of a TLR9 antagonist was accompanied by elevated viral gB RNA in the cornea (Figure 5I), confirming the critical role of TLR9 signaling in immune response during HSV-1 keratitis.

### Corneal pDCs Prevent Re-programming of Tregs to Effector Ex-Tregs in Acute HSV-1 Keratitis

To study the significance of pDCs in modulating adaptive immune responses, we assessed if pDCs affect the survival of Tregs, an important cellular player in ameliorating immune responses in HSV-1 keratitis (Suvas et al., 2004; Sehrawat et al., 2008; Veiga-Parga et al., 2012). Thus, we generated BM-chimeric mice, using WT C57BL/6 mice as recipients and mixture of BM cells from BDCA-2-DTR and Treg fate matting (FM) mice as donors, to concurrently deplete pDCs and assess the fate of Tregs. Confocal microscopy on corneal whole mounts of sham- and pDC-depleted chimeric mice on day 7 p.i. with HSV-1 indicted infiltration of both corneal Foxp3-eGFP^+^ Tdtomato^+^ Tregs (Figure 5J, white arrows), as well as Foxp3-eGFP^neg^ Tdtomato^+^ ex-Tregs. pDC depletion was accompanied by increased infiltrating Foxp3-eGFP^neg^ Tdtomato^+^ ex-Treg density compared with sham-depleted mice (Figures 5J and 5K). Next, we assessed ex-Treg density in dLNs to evaluate if the higher density of infiltrating corneal ex-Tregs upon pDC depletion is due to Treg re-programming in dLNs. After gating on live cells (Figures S5A and S5B), removing the doublets (Figures S5C and S5D), and gating on CD45^+^ CD4^+^ T cells (Figures S5E and S5F), we observed that corneal pDC depletion resulted in a 2.5-fold increase in the density of Foxp3-eGFP^neg^ Tdtomato^+^ ex-Tregs (Figure 5L), suggesting that pDC depletion facilitated re-programming of Foxp3-eGFP^+^ Tregs to Foxp3-eGFP^neg^ ex-Tregs in dLN. Next, phenotyping Foxp3-eGFP^neg^ ex-Tregs, we observed that in both control and pDC-depleted mice, the majority of Foxp3-eGFP^neg^ ex-Tregs expressed IFN-γ, suggesting their re-programming to effector T cells (Figure 5M). To assess if pDCs directly affect Treg survival, we next isolated splenic Tregs from Treg FM mice and cultured them with different densities of splenic pDCs sorted from WT C57BL/6 mice under stimulation of UV-irradiated HSV-1. On day 3 following co-culture, we performed immunofluorescence staining for PDCA-1 on the samples, followed by flow cytometry. After removing debris, dead cells, doublets, and pDCs (Figures S5G–S5O), we gated on Foxp3-eGFP^neg^ Tdtomato^+^ ex-Tregs and observed that whereas upon exposure to UV-irradiated HSV-1, Tregs convert to ex-Tregs, in the presence of pDC at densities of 1:10 and 1:1, pDCs considerably prevented the re-programming of Tregs to effector ex-Tregs (Figure 5N).

### Resident Corneal pDCs Mediate Corneal Immunity to Acute Primary HSV-1 Keratitis

**Figure 6.**
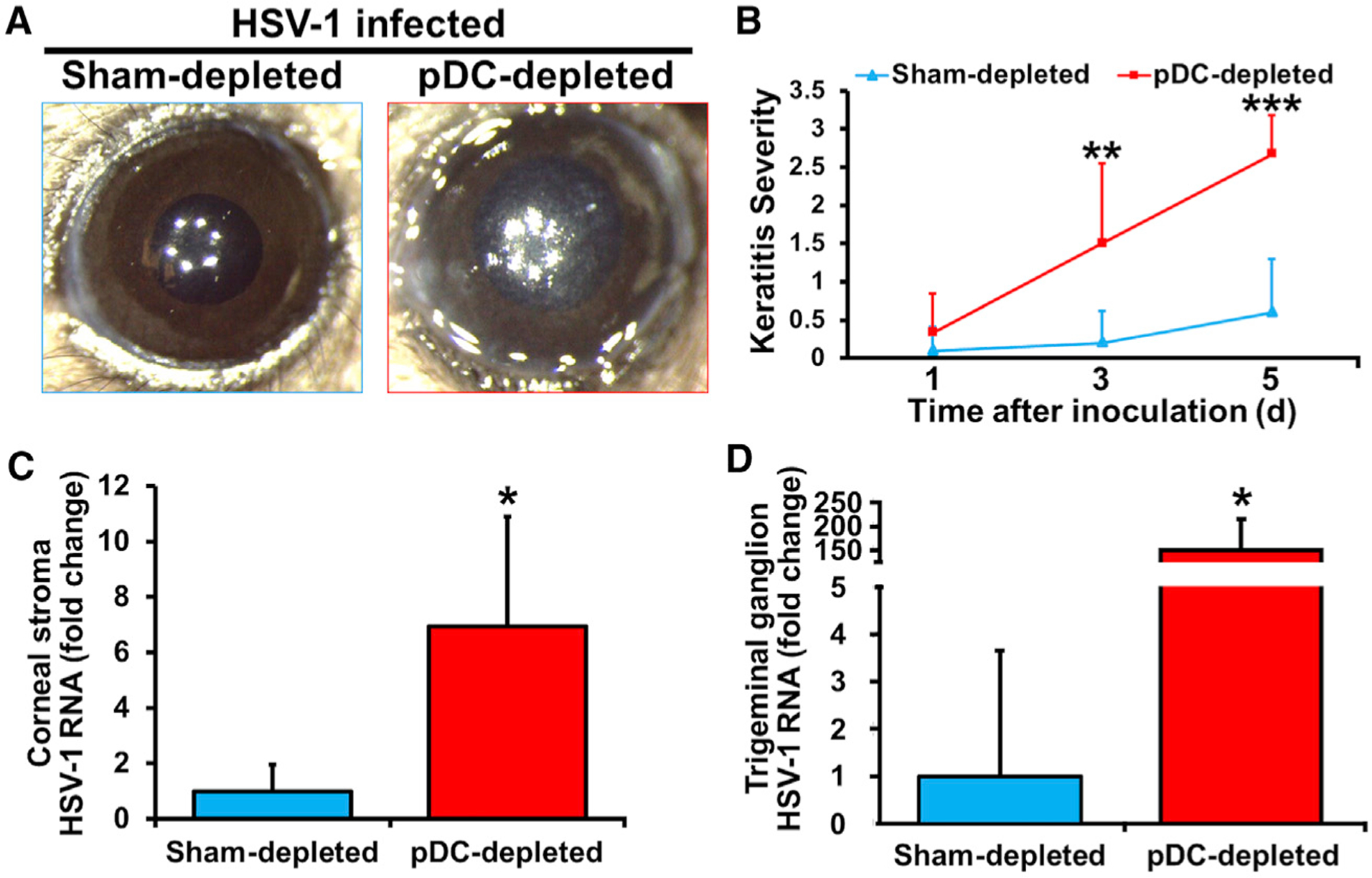
Presence of pDCs Prevents Corneal Manifestation following Inoculation with a Low Dose of HSV-1 but Does Not Totally Abolish Viral Transfer to the Trigeminal Ganglion (A and B) Representative clinical image of sham- and pDC-depleted corneas on day 5 following low-dose HSV-1 inoculation (A). Quantification of clinical severity of HSV-1 keratitis (n = 8–12/group) (B). (C and D) HSV-1 gB RNA in corneal stroma (C) and TG (D) of sham- and pDC-depleted corneas on day 5 following low-dose HSV-1 inoculation (n = 3 or 4/group). Bars denote SD. *p < 0.05, **p < 0.01, and ***p < 0.001.

Considering that in contrast to the clinical findings in the periocular skin (a tissue with no or sparse tissue-resident pDCs during steady state) (Wollenberg et al., 2002; Kohrgruber et al., 2004), clinical presentation of corneal involvement in primary HSV-1 infection is rare in humans (Darougar et al., 1985; Liesegang et al., 1989), we next tested if the lack of clinical findings during primary ocular HSV-1 infection may be attributed to the presence of tissue-resident corneal pDCs. Thus, we inoculated corneas of either sham- or pDC-depleted mice with a lower dose of HSV-1 (10^3^ plaque-forming units [PFU]), the peak amount of HSV-1 detected in murine tears following HSV-1 infection (Ghiasi et al., 2001). On day 3 after low-dose challenge, clinical assessment of corneas revealed a marked increase in the frequency of mice showing corneal opacification following pDC depletion (16.6% versus 83.3%; Figure 6A). On day 5 p.i., while corneal opacity was apparent in all pDC-depleted mice, only 25% of sham-depleted controls showed corneal opacity (Figure 6B). Furthermore, corneal pDC depletion was associated with enhanced viral dissemination to the corneal stroma and TG (Figures 6C and 6D), suggesting that the presence of corneal pDCs prevents the clinical presentation of primary HSV-1 infection in the cornea.

### Local Adoptive Transfer of pDCs Prevents Corneal Manifestations in Acute HSV-1 Keratitis

Observing the critical role of pDCs in minimizing the severity of corneal manifestations in acute HSV-1 keratitis, we next evaluated if local adoptive transfer of pDCs can diminish the clinical severity and enhance viral clearance. Thus, we adoptively transferred 10^4^ splenic pDCs 24 h prior to HSV inoculation to WT C57BL/6 mice. Adoptive transfer of pDCs was accompanied by decreased clinical severity on day 5 p.i. (Figures S6A and S6B). Assessment of corneal IFN-α level and viral load demonstrated that adoptive transfer of pDCs to corneas was associated with higher levels of IFN-α mRNA in the corneal stroma (Figure S6C) and lower viral gB RNA load (Figure 6D) in the cornea, suggesting enhanced viral clearance.

### Human Corneas Host Resident pDCs

To assess if our findings are clinically relevant to humans, we investigated if pDCs reside in naïve human corneas by performing flow cytometry in donor corneas. After gating out dead cells and debris, and gating on CD45^+^ cells (Figures S7A–S7C), we observed that similar to our findings in mice, approximately 18.7% of CD45^+^ cells expressed specific human pDC markers BDCA-2 and BDCA-4 (Figure S7D).

## DISCUSSION

### The Cornea Hosts a Resident Population of pDCs

To the best of our knowledge, this is the first comprehensive report on the constitutive presence, morphology, and distribution of tissue-resident pDCs in the cornea and their pivotal role in successfully combating corneal HSV-1 infection. Herein, we show that pDCs in the corneal barrier tissue play a pivotal protective role during acute HSV-1 infection by limiting viral replication, nerve damage, immune cell infiltration, and viral spread to extra-ocular tissues, contributing to attenuating symptoms of clinical keratitis and mortality. Furthermore, pDCs limit viral disease through direct TLR9-mediated IFN-α secretion and by preventing Treg re-programming to effector ex-Tregs. Thus, our findings suggest corneal pDCs may contribute to immunity of the cornea to primary HSV infection.

We demonstrate that in naïve corneas, CD45^+^ CD45R/B220^+^ PDCA-1^+^ pDCs are CD19^neg^ CD3^neg^ CD11b^neg^, indicating that they are distinct from B cells, T cells, or cDCs. Furthermore, their Ly6C, Ly49Q, and low CD11c expression emphasizes their phenotypical difference from previously identified stromal and epithelial cDCs in the cornea (Hamrah et al., 2002, 2003a, 2003b, 2003c). Identification of pDCs by TEM was based on the morphologic characteristics and localization of pDCs in the stroma, close to the epithelium. A limitation was lack of immuno-gold staining for pDC markers. We further observed presence of resident CD45^+^ BDCA-2^+^ BDCA-4^+^ pDCs in naïve human corneas. Although expression of these markers is considered a characteristic for pDCs, recent studies suggest that certain subpopulations of immune cells, such as AS-DCs, pre-cDCs, and transitional DCs, may express these markers as well (See et al., 2017; Villani et al., 2017; Leylek et al., 2019). These findings place the cornea among the very few non-lymphoid tissues in which resident pDCs have been reported, albeit in scarce numbers (Jameson et al., 2002; Bilsborough et al., 2003; Blasius et al., 2004; Omatsu et al., 2005; Lund et al., 2006). Our observation that the cornea, an immune-privileged barrier tissue, hosts pDCs during steady state, in light of the tolerogenic properties of pDCs (Goubier et al., 2008; Jongbloed et al., 2009; Rogers et al., 2013), may put forward possible roles of pDCs in the maintenance of immune privilege. Whether other immunologically privileged sites harbor pDCs during steady state, and if pDCs contribute to the preservation of immune privilege, deserve further studies.

**Figure 7.**
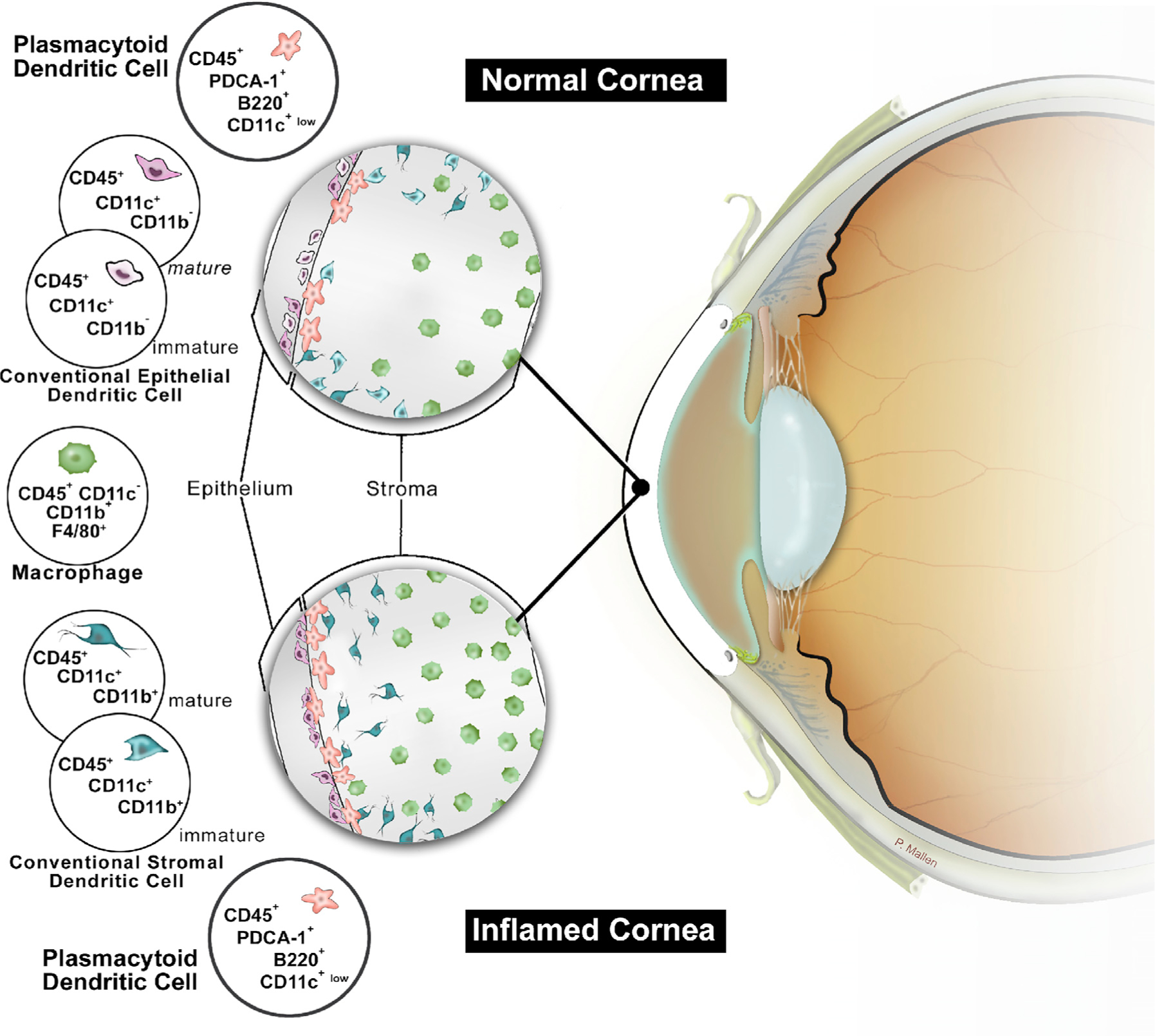
Schematic Diagram of Currently Identified Populations of Antigen-Presenting Cells in Naïve and Inflamed Corneas. Distribution of cDCs, macrophages, and pDCs in the anterior stroma of murine corneas under steady state as well as following corneal inflammation.

Previously, we and others have reported the presence of different subsets of APCs in the naïve cornea (Hamrah et al., 2002, 2003b, 2003c; Brissette-Storkus et al., 2002; Nakamura et al., 2005; Yamagami et al., 2005; Hamrah and Dana, 2007; Mayer et al., 2007). Our current novel discovery of resident corneal pDCs sheds additional light on the diversity of the corneal immune system, demonstrating the multiple layers of defense in this vital barrier organ that is needed to preserve vision (Figure 7). Using four-dimensional (4D) MPM, we demonstrated that pDC kinetics are significantly altered following inflammation, with pDCs becoming highly motile. The alterations in the migratory pDC kinetics may be mediated through chemokines or by induction of corneal edema during inflammation that may facilitate pDC movement.

### Protective Role of pDCs in HSV-1 Keratitis

Studies of pDCs have thus far been hampered by the lack of an easily accessible *in vivo* models whereby they can be studied under physiological and pathological conditions. Identification of resident corneal pDCs, a tissue that has been used to study biological processes, including angiogenesis, lymphangiogensis, and immune responses, as well as the advantage of local pDC depletion, can now facilitate *in vivo* studies of peripheral tissue pDCs.

We observed that pDC depletion leads to increased leukocyte recruitment to HSV-1 infected corneas. This in line with other groups’ findings that pDC depletion is associated with increased recruitment of cDCs and macrophages, secretion of pro-inflammatory cytokines, and higher viral load in the lungs and dLNs of mice infected with respiratory syncytial virus (Smit et al., 2006; Wang et al., 2006). Similarly, Soloff et al. (2012) showed that pDC depletion in mice with lethal influenza virus infection leads to considerable influx and activation of APCs. In contrast, Wolf et al. (2009) showed that Ikaros^l/l^ mice exhibit delayed increase in number of neutrophils and T cells in bronchoalveolar lavage of mice infected with influenza virus. However, this observation might be attributed to depletion of multiple other immune cells in addition to pDCs in this model.

Furthermore, Swiecki et al. (2013) reported that pDCs are important players in immune responses against systemic HSV-1 and HSV-2, but not local subcutaneous HSV-2 vaginal infection in mice. They observed that systemic depletion of pDCs does not affect viral load or IFN-α levels in the vagina of mice with local in-travaginal HSV-2 infection. However, depletion of pDCs was accompanied by decreased serum IFN-α level, natural killer (NK) cell activity, and mouse survival in systemic infection with HSV-1 or HSV-2 (Swiecki et al., 2013). In contrast, Yoneyama et al. (2005) showed that pDCs drive IFN-α production and promote antiviral cytotoxic T cell generation and viral clearance in the dLNs of mice with hind foot HSV-1 infection. Similarly, Vogel et al. (2014) showed that upon exposure to HSV-1, pDC-derived IFN-α activates NK cell activation *in vitro*. Taken together, these findings suggest that type and dose of pathogen, route of entry, and possibly pDC density at the entry site may affect antiviral responses to HSV infection. Current evidence on the role of local pDCs in the protection of a non-lymphoid barrier tissue against viral infection are scarce (Lund et al., 2006; Smit et al., 2006). Rather, it has been proposed that pDCs may represent a failsafe mechanism critical only for defense against systemic viral infections, once other mechanisms of protection in place at barrier tissues have been overwhelmed (Kumagai et al., 2007; Zucchini et al., 2008; Swiecki et al., 2013). Thus, our study provides important evidence on the direct and critical role of local pDC in the protection of a non-lymphoid tissue from viral infection.

### Significance of TLR9 and IFN-α in the Corneal Immune Response in HSV-1 Keratitis

Our experiments highlighted the essential role of TLR9 signaling for HSV-1-induced production of IFN-α in pDCs, as administration of TLR9 antagonist abolished the increase in the transcription of IFN-α. Studies in TLR9-knockout (KO) or IFN-α R-KO mice have demonstrated the critical role of these molecules for the induction of CXCL9 and CXCL10, the downstream recruitment of T cells and neutrophils in the cornea, and the control of viral shedding, highlighting the importance of IFN production in HSV keratitis (Conrady et al., 2011). Additionally, IFN-α has also been shown to limit the progress of infection from peripheral tissues to the nervous system (Halford et al., 1997). It has recently been shown that the corneal epithelium can produce IFN-α through TLR-dependent and TLR-independent innate sensor mechanisms (Hayashi et al., 2006; Kumar et al., 2006; Li et al., 2006; Conrady et al., 2012; Royer and Carr, 2016; Cui et al., 2017). Furthermore, recent studies have highlighted the importance of stimulator of IFN genes (STING), a cytoplasmic pathway of DNA recognition, in mediating immune responses to HSV-1. Interestingly, however, corneal inoculation with HSV-1 in STING-deficient mice had only a modest effect on type I IFN expression (Parker et al., 2015), further suggesting implications for other pathways of foreign DNA recognition, such as by endosomal TLRs during HSV-1 keratitis. Thus, although the key roles of TLR9 and IFN-α in HSV-1 keratitis and the secretion of IFN-α by the corneal epithelium are well established, the identity of TLR9^+^ cells and stromal sources of IFN-α in the cornea had remained elusive. Our discovery of a resident pDC population finally solves this mystery and clearly demonstrates the crucial role pDCs in HSV-1 keratitis.

Although the corneal epithelium contributes to IFN-α production during HSV-1 keratitis, high levels of IFN-α are observed in the corneal stroma (Conrady et al., 2011). We demonstrate that depletion of pDCs results in near total lack of IFN-α production, suggesting that pDCs are the main source of IFN-α in the corneal stroma. These data highlight the significance of pDCs and TLR9 signaling in IFN-α secretion during viral infections, as suggested by previous reports (Gurney et al., 2004; Krug et al., 2004; Smit et al., 2006; Wang et al., 2006; Kader et al., 2013; Schijf et al., 2013). Nevertheless, the signaling mechanism of TLR9 in corneal pDCs in HSV-1 keratitis needs to be further investigated. Although our observations suggest a critical role for TLR9 in the induction of IFN-α by pDCs, a recent study has shown that in MyD88 and TRIF-KO mice inoculated with a low dose (10^3^ PFU) of HSV-1, viral titers in the cornea were comparable with WT mice (Conrady et al., 2012). Thus, potential dose-dependent activation of diverse pathways of TLR signaling and IFN production such as potential MyD88-independent pathways needs to be considered in future studies.

### Impact of pDCs in Maintaining Functional Tregs in HSV-1 Keratitis

Tregs are vital modulators of adaptive immune responses to viral infections (Veiga-Parga et al., 2013), including HSV infections (Milman et al., 2016; Soerens et al., 2016). In HSV-1 keratitis, Treg depletion enhances disease severity via decreasing corneal macrophage and neutrophil recruitment (Veiga-Parga et al., 2012), effector T cell generation, activation, and migration to the cornea (Suvas et al., 2004; Veiga-Parga et al., 2012), and adoptive transfer of induced Tregs decreases clinical severity of the disease (Sehrawat et al., 2008). Recently, it has been shown that Tregs can acquire pathogenic effector T cell phenotype and secret IFN-γ, and their adoptive transfer can cause similar severity of keratitis compared with CD44^hi^ effector CD4 T cells (Bhela et al., 2017). Nevertheless, prior reports are controversial on the role of pDCs in induction of effector T cells over Tregs. Although earlier studies had suggested that pDC depletion leads to reduced generation of CD4^+^ T cells directly through IFN-α and decreased CD8^+^ T cells (Swiecki et al., 2010; Cervantes-Barragan et al., 2012), a recent study has suggested that pDC depletion accompanies reduced generation of neuropilin-1^+^ Tregs in viral challenges (Lynch et al., 2018). Our findings suggest that pDCs modulate adaptive T cell responses to HSV-1 keratitis, by directly promoting Treg survival and preventing Treg re-programming to effector ex-Tregs.

### Role of pDCs in Preventing Nerve Damage and Viral Dissemination

The observed higher rate of death upon pDC depletion in acute HSV keratitis can be explained by enhanced viral dissemination to extra-ocular tissues, resulting in the spread of the virus to the central nervous system and subsequent death. In contrast to pDCs, we recently demonstrated that depletion of cDCs in the cornea in HSV-1 keratitis is associated with decreased systemic spread of the virus to dLNs and TGs. cDCs also played an important role in limiting clinical keratitis and local corneal damage, but in contrast to pDCs they allowed systemic viral spread (Hu et al., 2015).

HSV keratitis is accompanied by nerve loss in both patients and murine HSV models (Hamrah et al., 2010; Cruzat et al., 2011; Yun et al., 2014; Chucair-Elliott et al., 2015). We show that although pDC depletion is associated with increased corneal nerve loss, it is also associated with increased neuronal invasion in the remaining nerves. In comparison, local depletion of cDCs, but not macrophages, in the cornea enhances subbasal nerve loss in HSV-1 keratitis; however, cDC depletion is accompanied by a decrease in neuronal invasion. Thus, although cDCs may facilitate infection of the nerve axons and transmission of the virus to the TG, pDCs prevent this process mainly by restricting the viral load. Although we have recently shown that corneal nerve loss in HSV keratitis can take place independent of leukocyte infiltration, Yun et al. (2014) demonstrated that depletion of CD4^+^ T cells limits corneal nerve damage in HSV keratitis, suggesting involvement of multiple contributors in the process of nerve damage. Thus, the mechanism of nerve damage and regeneration in HSV keratitis need to be further elucidated.

Recently, it has been shown that HSV-1 can directly infect the cornea primarily through the “front door” (Kaye et al., 1992; Kovacs et al., 2009; Shah et al., 2010), where it is then transmitted to the TG via sensory corneal nerves and remains latent. However, the reason why primary corneal infections do not typically result in clinical corneal manifestations has remained elusive (Darougar et al., 1985; Liesegang et al., 1989). Furthermore, although asymptomatic individuals shed the virus in their tears (Okinaga, 2000; Abiko et al., 2002; Khodadoost et al., 2004; Kaufman et al., 2005), it is not yet characterized how this persistent shedding does not result in more common HSV keratitis. We inoculated mouse corneas with a lower dose of HSV-1 and observed that while the low viral dose causes subtle clinical signs in a minority of pDC-sufficient mice, the majority of pDC-depleted corneas exhibit overt opacification. Nevertheless, consistent with prior reports, despite the lack of remarkable corneal manifestations in sham-depleted mice, HSV-1 could yet disseminate to corneal stroma and TG in both group of mice (LeBlanc et al., 1999). These findings suggest that lack of clinical findings in the cornea during primary HSV-1 infection may in part be explained by presence of tissue-resident pDCs, which minimize viral burden. Furthermore, local adoptive transfer of pDCs to cornea decreases the severity of clinical keratitis, increases IFN-α level in the corneal stroma, and accelerates viral clearance.

Collectively, we demonstrate that pDCs, a vital and distinct subset of BM-derived cells, reside in naïve corneas and play a unique role in viral elimination during HSV keratitis. Resident corneal pDCs contribute to immune responses against corneal HSV-1 infection by limiting viral load and dissemination by secretion of IFN-α through TLR9 signaling and promoting Treg survival. Further studies are paramount to reveal functions of pDCs in the cornea during homeostasis, as well as in other pathologic conditions.

## STAR★METHODS

### RESOURCE AVAILABILITY

#### Lead Contact

Further information and requests for resources and reagents should be directed to and will be fulfilled by the Lead Contact, Pedram Hamrah (pedram.hamrah@tufts.edu).

#### Materials Availability

All unique/stable reagents generated in this study are available from the Lead Contact.

#### Data and Code Availability

This study did not generate any unique datasets or code.

### EXPERIMENTAL MODEL AND SUBJECT DETAILS

#### Animals

Six- to ten-week-old male WT C57BL/6 (Charles River Laboratories International, Wilmington, MA), DPE-GFP × RAG-1^−/−^ (C57BL/6 background; kindly provided by Dr. Ulrich H. von Andrian, Harvard Medical School, Boston, MA) (Iparraguirre et al., 2008; Iannacone et al., 2010), BDCA2-DTR (C57BL/6 background; The Jackson Laboratory, Bar Harbor, ME; bred in house into homozygous) (Swiecki et al., 2010), CD11c-GFP-DTR mice (C57BL/6 background, The Jackson Laboratory; bred in house into homozygous) (Jung et al., 2002; Zhou et al., 2009), and Treg FM mice (generated by crossing Foxp3-eGFP/cre × Rosa-tdTomato mice; both from The Jackson Laboratory) (Zhou et al., 2009; Wang et al., 2012) were housed in specific pathogen-free conditions. BDCA2-DTR mice carry a transgene encoding a simian Diphtheria toxin receptor under the control of the human BDCA2 promoter. Murine pDCs do not express BDCA2, yet the BDCA2 promoter remains transcriptionally active in mice, which enables selective deletion of pDCs in these transgenic mice via exposure to DT (Swiecki et al., 2010). In Treg FM mice, eGFP and tdTomato are expressed under the control of the Foxp3 promoter. Thus, while Foxp3^+^ Tregs are Foxp3-eGFP^+^ tdTomato^+^, ex-Tregs which are Foxp3^neg^ are converted to Foxp3-eGFP^neg^ tdTomato^+^ cells (Zhou et al., 2009; Wang et al., 2012). All protocols were approved by the Harvard Medical School, Immune Disease Institute, Schepens Eye Research Institute, Tufts Medical Center, and Tufts University School of Medicine Animal Care and Use Committees. Animals were treated in accordance with the Association for Research in Vision and Ophthalmology Statement for the Use of Animals in Ophthalmic and Vision Research and NIH Guidelines for Animal Care.

#### Virus

The triple plaque-purified HSV-1 strain McKrae (kindly provided by Dr. Homayon Ghiasi, Cedars-Sinai Medical Center, Los Angeles, CA), a neurovirulent, stromal disease-causing strain, was used for ocular challenge (Sawtell et al., 1998; Ghiasi et al., 1999; Hu et al., 2015; Jiang et al., 2015). The McKrae HSV-1 strain was particularly chosen to enable survival studies, since less virulent strains, such as the KOS strain, generally do not cause mortality. For cell culture experiments, viruses were inactivated by exposure to 20 J UV light (15 minute [min] under biosafety cabinet UV transluminator; NuAire, Plymouth, MN) (Samudio et al., 2016).

#### Cells

Vero cells (derived from African green monkey kidney, kindly provided by Dr. Judy Lieberman, Children’s Hospital Boston, Boston, MA) were used to propagated HSV-1. Vero cells were cultured in DMEM and supplemented by 10% FBS. Primary cells were isolated from WT or transgenic mice per below.

#### Human Corneas

Human corneas were donated by Eversight (Ann Arbour, MI) in storage medium. Corneal single cell suspensions from 3 donors (with the age range of 25–64 years) were analyzed independently by flow cytometry within 7 days post mortem.

### METHOD DETAILS

#### Animal Procedures

For all of the procedures other than MPM, animals were anesthetized with intraperitoneal injections of 100 mg/kg Ketamine and 20 mg/kg Xylazine. For MPM, intraperitoneal injection of mixture of 100 mg/kg Ketamine, 20 mg/kg Xylazine, and 3 mg/kg acepro-mazine was used. Following corneal thermal burn or suture placement (see below), erythromycin ophthalmic ointment was applied on the eyes. After corneal debridement, thermal burn, suture placement, HSV inoculation, animals were treated with subcutaneous buprenorphine 0.05–0.1 mg/kg, every 8–12 h for 48 h to relieve post-surgical pain. Mice were randomly assigned to study groups using a Random Number Table.

#### Bone Marrow Chimeric Mice

WT C57BL/6 mice were irradiated twice with 600 rads with 3 hours (h) intervals, while their heads were covered with lead shielding to protect the eyes. 4 h later, their BMs were reconstituted by intravenous administration of 5 × 10^6^ BM cells obtained from femurs and tibias of BDCA2-DTR and Treg FM mice strains at a 2:1 ratio (Brown and Reiner, 2000; Allen et al., 2010). Chimeric mice were then housed for 4 weeks prior to further experimental procedures.

#### Corneal Thermal Burn as a Model for Acute Inflammation

Mice underwent corneal thermal cautery to induce acute inflammation as previously described (Williamson et al., 1987). Briefly, five light burns were performed on the central 50% of the cornea via a handheld thermal cautery (Aaron Medical Industries Inc., St. Petersburg, FL) under an operating microscope.

#### Corneal Suture Placement as a Model for Chronic Inflammation

Corneal suture placement was carried out to induce chronic inflammation, as previously described (Yamada et al., 1998). Briefly, three 11–0 nylon sutures (Sharpoint; Vanguard, Houston, TX) were placed through the paracentral stroma in the mid-peripheral cornea without perforating the cornea, using aseptic microsurgical technique and an operating microscope.

#### Acute HSV-1 Keratitis

Corneas were scarified 5 (horizontal) × 5 (vertical) times using a 30-gauge needle and topically inoculated with 10^3^ or 2 × 10^6^ PFU of HSV-1 strain McKrae in 10 μl DMEM culture media (Mediatech, Inc, Manassas, VA). Mice undergoing scarification and treatment with the same volume of virus-free DMEM constituted sham-infected controls.

#### Subconjunctival Injection

Local depletion of corneal pDCs was carried out via subconjunctival (subconj.) injections of 30 ng of DT (Sigma-Aldrich, St. Louis, MO) dissolved in 10 μl (5μl temporally and 5μl nasally) of phosphate buffered saline (PBS) in BDCA2-DTR or chimeric mice 2 days prior to HSV-1 inoculation, and repeated every 2 days thereafter in order to maintain continuous depletion (Hu et al., 2015). WT C57BL/6 mice treated with similar injections of DT or chimeric mice receiving PBS injections constituted the sham-controls. To block TLR9 activation, mice received subconj. injections of 10 mg oligonucleotide (ODN) 2088 (TLR9 antagonist; InvivoGen, San Diego, CA) or ODN 2088 Control (TLR9 antagonist control; InvivoGen) following HSV-1 inoculation with repeated injections every 48 h.

#### Ocular TLR9-Agonist Inoculation

Corneal epithelium of sham- or pDC-depleted mice were debrided using an Algerbrush II corneal rust ring (Alger Equipment Co, Lago Vista, TX) and either 20 μg phosphorothioate CpG 1826 oligonucleotide (CpG-ODN; a synthetic specific TLR-9 agonist; InvivoGen) or control oligonucleotide 1826 (Control ODN; InvivoGen) were topically administered on the eye.

#### Local Adoptive Transfer of pDCs

Following 24 h of culture, isolated splenic pDCs were resuspened in TISSEEL fibrin sealant (Baxter Healthcare Corporation, Deerfield, IL). Following debridement of the corneal epithelium using an Algerbrush II corneal rust ring, as above, 10^4^ pDCs diluted in the fibrin sealant were placed in the center of the cornea. Mice receiving fibrin sealant only served as controls.

#### Survival Studies

BDCA2-DTR or C57BL/6 WT mice received subconj. injections of DT, 2 days prior to inoculation of 2 × 10^6^ PFU of HSV-1. Subconj. injections were repeated every 48 h until day 6 p.i. (5 injections in total). Mice were monitored twice daily for survival for 20 days p.i. (n = 20/group). In addition to initial treatment after corneal scarification and viral inoculation, mice were given additional buprenorphine (0.1 mg/kg subcutaneously) to minimize suffering of any pain/distress (ocular swelling; red and discharge; inactivity; lack of food or water intake; changes in gait), if needed. In case of suffering from severe pain/distress (ruffled fur; hunched posture; crouching; shivering), mice were euthanized and were counted as endpoints (death).

#### Clinical Evaluation of Herpes Simplex Keratitis Severity

The severity of acute keratitis was assessed by a blinded observer by slit-lamp bio-microscopy as previously described (Inoue et al., 2000; Hu et al., 2015). Briefly, corneal opacification was scored using the following scoring: 0, normal; 1, corneal opacity confined to less than one quarter of the cornea with visible iris; 2, corneal opacity between one quarter and one half of the cornea with visible iris; 3, corneal opacity extended to greater than half of the cornea with partially invisible iris; and 4, maximal corneal opacity spread over the entire cornea and completely invisible iris.

#### Antibodies

The following antibodies (Abs) were obtained from BD Biosciences (San Jose, CA), eBioscience (San Diego, CA), BioLegend (San Diego, CA), or Miltenyi Biotec, (Bergisch Gladbach, Germany) unless otherwise noted: fluorochrome-labeled Ab (clone) against CD45 (30-F11 and HI30), PDCA-1 (927 and JF05–1C2.4.1), CD45R/B220 (RA3–6B2), Siglec-H (eBio440c), CD11c (HL3), Ly6C (HK1.4), Ly49Q (clone number 2000000; MBL International Corporation, Woburn, MA), Gr-1 (RB6–8C5), Ly6G (1A8), CD11b (M1/70), CD68 (FA-11), CD4 (RM4–5), CD3 (17A2), CD19 (6D5), IFN-γ (XMG1.2), F4/80 (BM8), BDCA-2 (201A), BDCA-4 (12C2), β-III-Tubulin (TuJ-1; R&D Systems, Minneapolis, MN), and HSV-1 (polyclonal; Dako, Carpinteria, CA). Fluorochrome-conjugated rat IgG1, IgG2a, IgG2b, IgG2c, mouse IgG1, IgG2a, and Armenian hamster IgG1 were used as isotype-matched controls.

#### Single Cell Suspension and Flow Cytometry

TGs, single human and pooled mice corneas were cut into pieces and digested via incubation with 2 mg/ml collagenase D (Roche, Indianapolis, IN) and 0.05 mg/ml DNase (Roche) to yield single cell suspensions. BM and dLN samples were filtered and underwent RBC lysis via incubation with Ammonium Chloride Potassium (ACK) RBC lysis buffer (Biofluids, Rockville, MD). LIVE/DEAD Fixable Blue Dead Cell Stain kit, for UV (Thermo Fisher Scientific, Waltham, MA) was used to assess viable cells in murine corneal single cell suspensions. After blocking for 15 min with 1% anti-CD16/CD32 Fc receptor (FcR) mAb (2.4G2; Bio × Cell, West Lebanon, NH) in 0.5% bovine serum albumin (BSA; Sigma-Aldrich) at 4°C, samples were labeled with combinations of Abs or their respective isotype controls. Intracellular staining of IFN-γ was performed using the Fixation/Permeabilization Solution Kit with BD GolgiPlug (BD Biosciences) according to the manufacturer’s instructions. Samples were then washed and analyzed with a BD LSR II flow cytometer (BD Biosciences). Data were analyzed with FlowJo V9.2 (FlowJo, LLC, Ashland, OR). Forward and side scatterplots were used to exclude dead cells, debris, and doublets. For each experiment on mice corneas, 15–20 control corneas (naïve or sham-infected) or 4–8 inflamed (thermal cautery, suture placed, or HSV-1-infected) corneas were pooled.

#### Cell Sorting

For single cell PCR, the following cells were sorted: (1) corneal GFP-tagged pDCs from pooled (n = 10) collagenase-digested naïve corneas of DPE-GFP × RAG-1^−/−^ mice using WT C57BL/6 mice as controls for GFP sorting; (2) splenic pDCs from naïve WTC57BL/6 mice; and (3) splenic macrophages from naïve WT C57BL/6 mice. For Treg survival assays, splenic Tregs from naïve Treg FM mice and splenic pDCs from naïve WT C57BL/6 mice were sorted. For pDC culture and adoptive transfer studies, splenic GFP^+^ pDCs were sorted from DPE-GFP × RAG-1^−/−^ mice. Briefly, spleens were harvested, mechanically dissociated and passed through a 40 μm cell strainer (BD Falcon) to yield single cell suspensions of splenic cells. Next, RBCs were lysed using ACK RBC lysis buffer. For isolating pDCs and macrophages from WT C56BL/6 mouse for single cell PCR experiments, splenocytes were stained with PDCA-1, CD45R/B220, Siglec-H, and F4/80 or their respective isotype controls. Triple-positive PDCA-1^+^ CD45R/B220^+^ Siglec-H^+^ pDCs and single positive F4/80^+^ macrophages were sorted. For Treg survival assays, PDCA-1^+^ CD45R/B220^+^ pDCs were sorted. All sortings were performed using MoFlo Astrios EQ (Beckman Coulter).

#### Immunofluorescence Staining and Confocal Microscopy

For immunofluorescence staining, corneas were excised and the epithelium was removed, as previously described (Hamrah et al., 2002). Briefly, freshly excised corneas were immersed in PBS, containing 20 mM EDTA (Sigma-Aldrich) at 37°C for 30 min. Subsequently, the epithelium was removed with forceps and the stroma was washed in PBS. Samples were fixed in chilled acetone (Sigma-Aldrich) at room temperature (RT), and following three washes, were blocked in 2% BSA and 1% anti-CD16/CD32 FcR mAb (Bio × Cell) for 30 min at RT, and incubated with combinations of primary Abs including CD45, PDCA-1, CD45R/B220, β-III-Tubulin, and HSV-1 or isotype controls overnight at 4°C or at RT for 2 h. After washings, samples were mounted with DAPI-containing medium (Vector Laboratories Inc., Burlingame, CA) and imaged by confocal microscopy (Fluoview BX50WI microscope [Olympus, Japan] or a Leica TCS SP8 [Leica Microsystems, Wetzlar, Germany]). Corneas excised from chimeric mice were fixed in 4% PFA and corneas from DPE-GFP × RAG-1^−/−^ mice were visualized freshly by either a Fluoview BX50WI or a Leica TCS SP8 microscope. Central and peripheral areas for each cornea were assessed separately, as previously described (Hamrah et al., 2002). Cell densities were quantified via IMARIS (Bitplane AG, Zurich, Switzerland) and quantification of corneal nerve density was performed using NeuronJ plugin (Meijering et al., 2004) for ImageJ software (NIH, Bethesda, MD) (Hu et al., 2015). To measure the percentage of infected nerves, double stained (β-III-Tubulin^+^ HSV-1^+^) nerve segments were considered as infected and their lengths were divided by the total nerve length.

#### pDC Culture

10^5^ sorted splenic pDCs from DPE-GFP × RAG-1^−/−^ mice (as described above) were seeded in 48-well plates and cultured with DMEM supplemented with 10% fetal bovine serum (FBS; Gemini Bioproducts, Woodland, CA), and 1% penicillin/streptomycin (Life Technologies, Carlsbad, CA). Cells were treated with 1 μg/ml Control ODN, 1 mg/ml CpG-ODN and 10 mg/ml ODN 2088 Control, 1 μg/ml CpG-ODN and 10 μg/ml ODN 2088,10^5^ PFU UV-irradiated McKrae HSV-1 and 10 μg/ml ODN 2088 Control, or 10^5^ PFU UV-inactivated McKrae HSV-1 and 10 μg/ml ODN 2088. Cells were harvested 24 h after culture for analysis.

#### Treg Survival Assay

10^5^ sorted splenic Tregs were sorted from Treg FM mice and were cultured with different densities of sorted splenic pDCs from WT C57BL/6 mice (at 1:0, 1:10, and 1:1 Treg:pDC ratios) under treatment with with 10^5^ PFU UV- irradiated McKrae HSV-1 for 3 days in DMEM, supplemented with 10% fetal bovine serum, 1% penicillin/streptomycin, and 1 ng/ml TGF-β1 (eBioscience). Cells then underwent immunofluorescence staining for PDCA-1 followed by flow cytometry (as above).

#### Multi-photon Microscopy

An upright commercial Ultima two-photon microscope (Bruker Corporation, Billerica, MA) equipped with tunable Mai Tai Ti:sapphire lasers (Spectra-Physics/Newport Company, Santa Clara, CA) was used for multiphoton excitation. MPM was performed on freshly excised, unstained and unfixed corneas of DPE-GFP × RAG-1^−/−^ or CD11c-GFP-DTR mice at an excitation wavelength of 880 nm. For 3D analysis, stacks of 60 optical x-y sections with 1-μm z spacing were acquired with electronic zooming to 1.0–3.0 × through a 20 × /0.95 numerical aperture water-immersion objective lens (Olympus, Center Valley, CA). Emitted fluorescence and second-harmonic signals were detected through 400/40 nm, 450/80 nm, 525/50 nm and 630/120 nm bandpass filters with non-descanned detectors to generate multi-color images. For assessing kinetics, stacks of multiple x-y sections with 3 μm z spacing were acquired every 60 s for at least 30 min to provide image volumes of at least 130 μm in depth. Three-dimensional reconstruction of z stacks was performed by Volocity (PerkinElmer, Waltham, MA). IMARIS (Bitplane AG) was used for 4D image analysis and movie production. Background subtraction, noise reduction, brightness/contrast adjustment, compression and size adjustment were primarily performed and the files were rendered as maximum-intensity projection providing a realistic 4D movie. GFP^+^ cells were pointed as individual spots and corneal collagen was used for delineation of stroma. The following kinetic parameters were generated: 3D mean speed (μm/min), track length (total distance traveled by a single spot, μm), track displacement length (the shortest length connecting initial and final spatial location of a single spot, μm), meandering index measured by dividing displacement length by total track length. Presented track displacements were adjusted to reflect cell displacement in 1 h 4D movies.

#### Transmission Electron Microscopy

WT C57BL/6 corneas were fixed in Karnovsky solution. After three washes in cacodylate buffer, samples were post-fixed in 1% osmium tetroxide for 1.5 h. Next, samples were washed with water, stained in aqueous 2% uranyl acetate, dehydrated, and embedded in Epon. Samples were sectioned at 6 nm and imaged by a transmission electron microscope (410 TEM; Philips, Eindhoven, Netherlands).

#### Corneal Viral Titers

Corneas were homogenized using gentleMACS Dissociator (Miltenyi Biotec) and HSV-1 viral load was determined by standard virus plaque assay on Vero cells. In brief, 100 mL serial dilutions of corneal homogenates were plated on Vero cell monolayers cultured in 6-well plates and incubated at 37°C for 1 h. Monolayers were rinsed, overlaid with 0.5% methylcellulose (Sigma-Aldrich) in DMEM supplemented with 5% FCS, and incubated at 37°C for 2–3 days. Subsequently, plates were stained with 1% crystal violet (Sigma-Aldrich) and plaques were counted.

#### RNA Isolation, cDNA Synthesis, and Quantitative Real-time PCR

For experiments with application of ODNs in mice and adoptive transfer of pDCs, the corneal epithelium was separated from the stroma as described above. RNA was extracted from TGs, dLNs, whole corneas, and corneal stromas with RNeasy Plus Universal Mini kit (QIAGEN, Germantown, MD). For *in vitro* experiments, cells were collected after 24-h culture and RNA was extracted via SingleShot Cell Lysis (Bio-Rad Laboratories, Hercules, CA). cDNA was synthetized using iScript cDNA synthesis kit (Bio-Rad Laboratories). For samples obtained from *in vitro* culture and adoptive transfer studies, cDNA was pre-amplified using SsoAdvanced PreAmp Supermix (Bio-Rad Laboratories) according to the manufacturer’s instructions. For single cell qRT-PCR, 100 corneal GFP^+^ pDCs, naïve splenic pDCs, and macrophages were lyzed via REPLI-g Cell WGA & WTA kit (QIAGEN) according to manufacturer’s instructions. qRT-PCR was carried out in triplicates using the SYBR Premix EX TaqII (Takara, Japan) or SsoAdvanced Universal SYBR Green Supermix (Bio-Rad Laboratories), and analyzed using a Bio-Rad iCycler iQ thermocycler (Bio-Rad Laboratories). The sequence of primers used is available in Table S1. Relative fold changes are reported using delta-delta cycle threshold (ΔΔCt) method.

#### Corneal ELISA

For experiments using ODN, the epithelium was removed. Whole corneas and corneal stromas were lysed in tissue protein extraction reagent T-PER (Thermo Fisher Scientific) and homogenized by gentleMACS Dissociator (Miltenyi Biotec). IFN-α protein levels were measured via mouse IFN-α ELISA kit (BMS6027; eBioscience).

### QUANTIFICATION AND STATISTICAL ANALYSIS

Statistical analysis was performed using SPSS 16.0 (SPSS Inc., Chicago, IL). Student’s t test was used for comparing means between two groups and one-way ANOVA with Bonferroni’s or LSD post hoc test was used for comparisons among three or more groups, where appropriate. Chi-square test was used for comparing qualitative variables. Kaplan-Meier curve with log-rank test was used in survival analysis. The number of experiments and mice are indicated in the individual Figure Legends. p < 0.05 was considered statistically significant.

## Supplementary Material

1

2

3

4

5

6

7

8

## Figures and Tables

**Table:** KEY RESOURCES TABLE

REAGENT or RESOURCE	SOURCE	DENTIFIER
Antibodies		
anti-CD16/CD32 Fc receptor (FcR) mAb (2.4G2)	Bio × Cell	Cat. # BE0307; RRID: AB_2736987
Anti-mouse CD45 (30-F11)	BioLegend	Cat. # 103106; RRID: AB_312971
Anti-mouse CD45 (30-F11)	BioLegend	Cat. # 103124; RRID: AB_493533
Anti-mouse CD45 (30-F11)	BioLegend	Cat. # 103126; RRID: AB_493535
Anti-mouse CD45 (30-F11)	BioLegend	Cat. # 103108; RRID: AB_312973
Anti-human CD45 (HI30)	BioLegend	Cat. # 304005; RRID: AB_314393
Anti-mouse PDCA-1 (JF05-1C2.4.1)	Miltenyi Biotech	Cat. # 130-102-260; RRID: AB_2659966
Anti-mouse PDCA-1 (927)	BioLegend	Cat. # 127010; RRID: AB_1953285
Anti-mouse PDCA-1 (927)	BioLegend	Cat. # 127014; RRID: AB_1953289
Anti-mouse/human CD45R/B220 (RA3-6B2)	BioLegend	Cat. # 103222; RRID: AB_313005
Anti-mouse/human CD45R/B220 (RA3-6B2)	BioLegend	Cat. # 103205; RRID: AB_312990
Anti-mouse Siglec-H (eBio440c)	eBioscience	Cat. # 11-0333-82; RRID: AB_837163
Anti-mouse F4/80 (BM8)	BioLegend	Cat. # 123120; RRID: AB_893479
Anti-mouse F4/80 (BM8)	BioLegend	Cat. # 123123; RRID: AB_893487
Anti-mouse CD11c (HL3)	BD Biosciences	Cat. # 561045; RRID: AB_10562385
Anti-mouse Ly6C (HK1.4)	BioLegend	Cat. # 128011; RRID: AB_1659242
Anti-mouse Gr-1 (RB6-8C5)	BioLegend	Cat. # 108428; RRID: AB_893558
Anti-mouse Gr-1 (RB6-8C5)	BioLegend	Cat. # 108423; RRID: AB_2137486
Anti-mouse Ly6G (1A8)	BioLegend	Cat. # 127616; RRID: AB_1877271
Anti-mouse Ly49Q (clone number 2000000)	MBL International Corporation,	Cat. # D160-4; RRID: AB_592121
Anti-mouse/human CD11b (M1/70)	BD Biosciences	Cat. # 553310; RRID: AB_394774
Anti-mouse CD68 (FA-11)	BioLegend	Cat. # 137015; RRID: AB_2562947
Anti-mouse CD4 (RM4-5)	BioLegend	Cat. # 100530; RRID: AB_389325
Anti-mouse CD3 (17A2)	BioLegend	Cat. # 100237; RRID: AB_2562039
Anti-mouse CD19 (6D5)	BioLegend	Cat. # 115539; RRID: AB_11203538
Anti-mouse IFN-γ (XMG1.2)	BioLegend	Cat. # 505821; RRID: AB_961361
Anti-human BDCA-2 (201A)	BioLegend	Cat. # 354217; RRID: AB_2571982
Anti-human BDCA-4 (12C2)	BioLegend	Cat. # 354507; RRID: AB_2561556
Anti-mouse β-III-Tubulin (TuJ-1)	R&D Systems	Cat. # NL1195V; RRID: AB_1241877
Anti- HSV-1 (polyclonal)	Dako	Cat. # F0318; This antibody is no longer available. The only one that is available from Dako/Agilent is a unconjugated, concentrated form. The available antibody details are here: https://www.agilent.com/store/productDetail.jsp?catalogId=B011402-2
Rat IgG1, κ isotype control	BioLegend	Cat. # 400425; RRID: AB_893689
Rat IgG2b, κ isotype control	BioLegend	Cat. # 400608; RRID: AB_326552
Rat IgG2b, κ isotype control	BioLegend	Cat. # 400627; RRID: AB_493561
Rat IgG2b, κ isotype control	BioLegend	Cat. # 400626; RRID: AB_389343
Rat IgG2a, κ isotype control	BioLegend	Cat. # 400522; RRID: AB_326542
Armenian hamster IgG1,λ2 isotype control	BD Biosciences	Cat. # 553953; RRID: AB_395157
Rat IgG2c, κ isotype control	BiolLgend	Cat. # 400723; RRID: AB_2864281
Rat IgG2b, κ isotype control	BioLegend	Cat. # 400631; RRID: AB_893693
Rat IgG2b, κ isotype control	BD Biosciences	Cat. # 553988; RRID: AB_479619
Rat IgG2b, κ isotype control	BioLegend	Cat. # 400649; RRID: AB_2864282
Rat IgG2a, κ isotype control	BioLegend	Cat. # 400525; RRID: AB_2864283
Rat IgG2a, κ isotype control	BioLegend	Cat. # 400526; RRID: AB_2864284
Rat IgG2a, κ isotype control	BioLegend	Cat. # 400527; RRID: AB_2864285
Rat IgG2a, κ isotype control	BioLegend	Cat. # 400531; RRID: AB_2864286
Rat IgG2a, κ isotype control	BioLegend	Cat. # 400539; RRID: AB_11126979
Rat IgG2b, κ isotype control	BioLegend	Cat. # 400623; RRID: AB_326565
Rat IgG2b, κ isotype control	eBioscience	Cat. # 17-4031-81; RRID: AB_470175
Rat IgG2a, κ isotype control	BioLegend	Cat. # 400505; RRID: AB_2736919
Mouse IgG1, κ isotype control	BioLegend	Cat. # 400107; RRID: AB_326429
Mouse IgG2a, κ isotype control	BioLegend	Cat. # 400234; RRID: AB_2864287
Mouse IgG2a, κ isotype control	BioLegend	Cat. # 400231; RRID: AB_2864288
Bacterial and Virus Strains		
HSV-1 strain McKrae	Gift from Dr. H. Ghiasi	(Mott et al., 2007)
Biological Samples		
Human corneas	Eversight	N/A
Chemicals, Peptides, and Recombinant Proteins		
LIVE/DEAD Fixable Blue Dead Cell Stain kit, for UV	Thermo Fisher Scientific	Cat. # L34961
Diphtheria toxin	Sigma-Aldrich	Cat. # D0564-1MG
TISSEEL fibrin sealant	Baxter Healthcare Corporation	Cat. # 1506079
Collagenase D	Roche	Cat. # 11088866001
DNase	Roche	Cat. # 10104159001
Cytofix/Cytoperm Fixation/Permeabilization Solution Kit with BD GolgiPlug	BD Biosciences	Cat. # 555028
TGF-β1	eBioscience	Cat. # 14-8342-62
Methylcellulose	Sigma-Aldrich	Cat. # M0512-100G
Crystal violet	Sigma-Aldrich	Cat. # C3886-25G
Ethylenediaminetetraacetic acid (EDTA) disodium salt solution Disodium Salt	Sigma-Aldrich	Cat. # E7889
Critical Commercial Assays		
IFN-α ELISA kit	eBioscience	Cat. # BMS6027
RNeasy Plus Universal Mini kit	QIAGEN	Cat. # 73404
SingleShot Cell Lysis kit	Bio-Rad Laboratories	Cat. # 1725080
iScript cDNA synthesis kit	Bio-Rad Laboratories	Cat. # 1708891
SsoAdvanced PreAmp Supermix	Bio-Rad Laboratories	Cat. # 1725160
REPLI-g Cell WGA & WTA kit	QIAGEN	Cat. # 150052
SsoAdvanced Universal SYBR Green Supermix	Bio-Rad Laboratories	Cat. # 1725272
SYBR Premix EX TaqII	Takara	Cat. # RR081A
Tissue protein extraction reagent (T-PER)	Thermo Fisher Scientific	Cat. # 78510
gentleMACS Dissociator M Tubes	Miltenyi Biotec	130-093-236
Experimental Models: Cell Lines		
Vero cells	Gift from Dr. J. Lieberman	(Palliser et al., 2006)
Experimental Models: Organisms/Strains		
DPE-GFP × RAG-1^−/−^	Gift from Dr. U. von Andrian	(Iparraguirre et al., 2008)
BDCA2-DTR	The Jackson Laboratory	Stock #: 014176; IMSR_JAX:014176
CD11c-GFP-DTR	The Jackson Laboratory	Stock #: 004509; IMSR_JAX:004509
Foxp3-eGFP/cre	The Jackson Laboratory	Stock #: 023161; IMSR_JAX:023161
Rosa-tdTomato	The Jackson Laboratory	Stock #: 007914; IMSR_JAX:007914
WT C57BL/6	Charles River Laboratories International	Strain Code # 027; IMSR_CRL:027
Oligonucleotides		
Oligonucleotide 2088	InvivoGen	Cat. # tlrl-2088
Oligonucleotide 2088 Control	InvivoGen	Cat. # tlrl-2088c
Phosphorothioate CpG 1826 oligonucleotide	InvivoGen	Cat. # tlrl-1826
Control oligonucleotide 1826	InvivoGen	Cat. # tlrl-1826c
Primers for qRT-PCR see Table S1	Integrated DNA Technologies, Inc.	N/A
Software and Algorithms		
FlowJo v9.2	FlowJo LLC.	https://www.flowjo.com/; SCR_008520
ImageJ	(Schneider et al., 2012)	https://imagej.nih.gov/ij/; SCR_003070
NeuronJ	(Meijering et al., 2004)	https://imagescience.org/meijering/software/neuronj/; SCR_002074
Volocity	PerkinElmer	https://www.perkinelmer.com; SCR_002668
IMARIS	Bitplane AG	https://imaris.oxinst.com/; SCR_007370
SPSS 16.0	SPSS Inc.	Current company: https://www.ibm.com/analytics/spss-statistics-software; SCR_002865

## Highlights

- The cornea, as a peripheral barrier, hosts resident pDCs during steady state
- Local pDC depletion enhances HSV-1 keratitis severity, nerve damage, and viral spread
- pDCs secrete IFN-α via TLR9 and preserve Tregs in the dLNs in acute HSV-1 keratitis
- pDCs may protect the cornea from clinical signs of primary HSV-1 corneal infection
