# Supplementary material for: Characterization of Resident Corneal Plasmacytoid Dendritic Cells and Their Pivotal Role in Herpes Simplex Keratitis
Source: Cell Rep. Author manuscript; Available in PMC 2020 Sep 23. (PMC7511260; doi:10.1016/j.celrep.2020.108099)
Supplement: 1 [file NIHMS1627852-supplement-1.pdf]

Cell Reports, Volume *32*

Supplemental Information

Characterization of Resident Corneal Plasmacytoid Dendritic Cells and Their Pivotal Role in Herpes Simplex Keratitis

Arsia Jamali, Kai Hu, Victor G. Sendra, Tomas Blanco, Maria J. Lopez, Gustavo Ortiz, Yureeda Qazi, Lixin Zheng, Aslihan Turhan, Deshea L. Harris, and Pedram Hamrah

Supplementary Figure 1 (related to Figure 1)

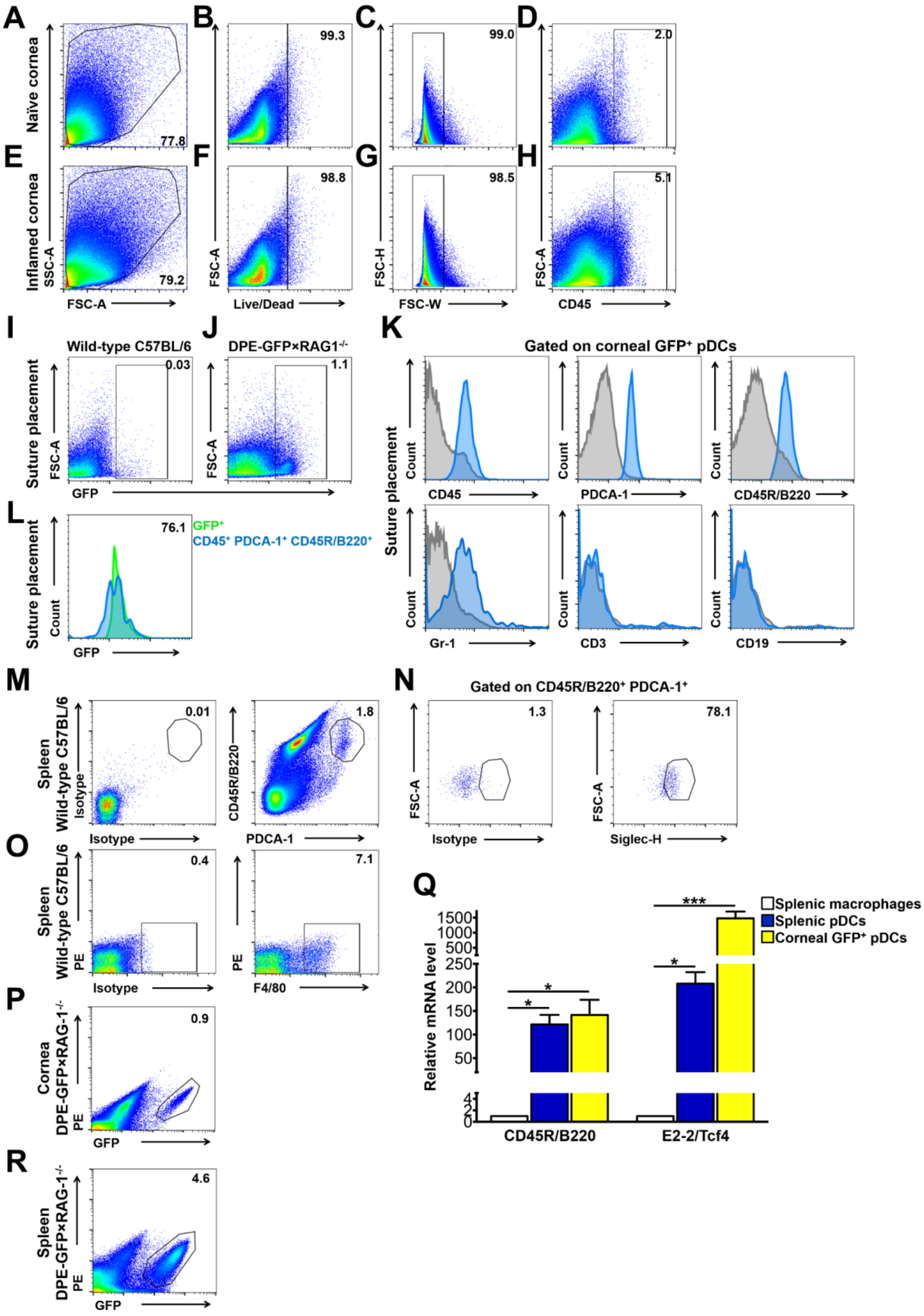

Figure S1. Flow Cytometry Analysis and Sorting Gating Strategies and Characterization of GFP-tagged Cells in the Cornea of DPE-GFP×RAG-1$^{-/-}$Mice (related to Figure 1)**.**

(**A-H**) Flow cytometry gating strategies for naïve (**A-H**) and cautery-induced inflamed (**E-H**) WT C57BL/6 corneas. Gating strategy for removing debris (**A** and **E**), dead cells (**B** and **F**), and doublets (**C** and **G**), followed by gating on CD45^{+} cells (**D** and **H**).

(**I-L**) Characterization of GFP^{+} cells in the cornea of DPE-GFP×RAG-1$^{-/-}$ mice by flow cytometry. (**I-J**) Representative dot plots of single cell suspension of digested sutured corneas of WT C57BL/6 mice (used as controls; **I**) and DPE-GFP×RAG-1$^{-/-}$ mice, showing GFP^{+} cells in the inflamed corneas (**J**). (**K**) Flow cytometry histograms on expression of CD45, PDCA-1, CD45R/B220, Gr-1, CD3, and CD19 by GFP^{+} cells. Isotype controls are shown in grey; blue histograms reflect staining of target molecules. (**L**) Flow cytometry histogram of CD45^{+} PDCA-1^{+} CD45R/B220^{+} Gr-1^{+} F4/80^{+} pDCs (blue) and GFP^{+} cells (green) in the cornea of DPE-GFP×RAG-1$^{-/-}$ mice on day 7 following suture placement.

(**M-P**) Flow cytometry dot plots representing sorting splenic pDCs (**M-N**) and macrophages (**O**) from naïve WT C57BL/6 mice and corneal pDCs of DPE-GFP×RAG-1$^{-/-}$ mice (**P**). Selecting splenic PDCA-1^{+} CD45R/B220^{+} cells (**M**; right panel) using respective isotype controls as guides, after initial gating out debris and doubles (**M**, left panel); Further sorting for Siglec-H^{+} cells (**N**) to yield triple positive PDCA-1^{+} CD45R/B220^{+} Siglec-H^{+} splenic pDCs. Sorting splenic macrophages by gating on F4/80^{+} cells (**O**; left panel), using isotype control and PE channel to exclude non-macrophages (**O**, right panel). Sorting corneal GFP^{+} pDCs from digested sutured corneas of naïve DPE-GFP×RAG-1$^{-/-}$ mice by primary gating out debris and doublets followed by sorting GFP^{+} cells using PE channel as guide to exclude auto-fluorescent signals (**P**).

(**Q**) Single cell qRT-PCR of pDC markers E2-2/Tcf4 and CD45R/B220 in splenic triple PDCA-1^+ CD45R/$B220^+$ Siglec-H^+ pDCs, GFP^+ corneal pDCs, and splenic macrophages.

(**R**) Representative flow cytometry dot plot showing sorting strategy of splenic GFP^+ pDCs of DPE-GFP×RAG-$1^{-/-}$ mice following gating out debris and doublets.

Flow cytometry analysis plots are representative of three independent experiments. Bars denote SD. * $p < 0.05$, *** $p<0.001$

Supplementary Figure 2 (related to Figure 1)

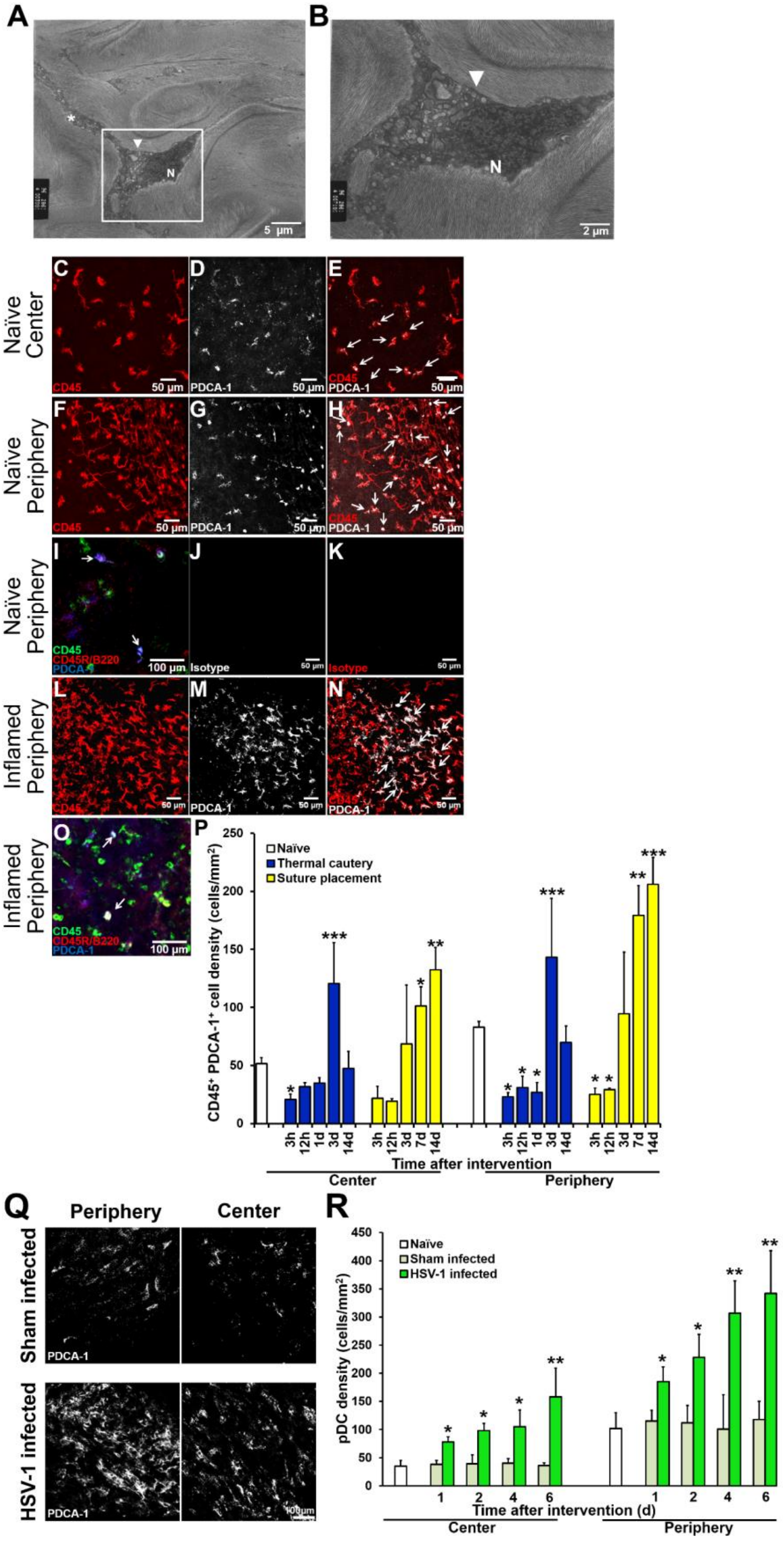

Figure S2. Characteristics and Distribution of pDCs in Naïve and Inflamed Murine Corneas (related to Figure 1).

(**A, B**) Transmission electron micrograph of a resident pDC in naïve WT C57BL/6 mouse cornea (**A**). Inset (cell body) is magnified in (**B**). An eccentric nucleus (**N**) with predominant heterochromatin is apparent in the cell body (**white arrowhead**). Also, numerous vacuole-like endosomes, lysosomes, or other vesicles are present. One thick, stub-like process is marked (**white asterisk**).

(**C-H**) Representative double staining of corneal whole-mount of a naïve WT C57BL/6 mouse with CD45 (**C** and **F**) and PDCA-1 (**D** and **G**) illustrating $CD45^+$ PDCA-1^+ cells (**E** and **H**; arrows) in the central (**C**-**E**) and peripheral (**F-H**) cornea.

(**I**) Representative triple-staining of a naïve cornea of WT C57BL/6 mouse with CD45, PDCA-1, and CD45R/B220. The arrow indicates $CD45^+$ PDCA-1^+ CD45R/$B220^+$ cells in the corneal periphery (arrow).

(**J**-**K**) Representative isotype control staining for PDCA-1 (**J**) and CD45R/B220 (**K**).

(**L-N**) Representative double-staining of corneal whole-mount in a WT C57BL/6 mouse on day 3 following thermal cautery with CD45 (**L**) and PDCA-1 (**M**) illustrating $CD45^+$ PDCA-1^+ cells (**N**; arrows) in the peripheral cornea.

(**O**) Representative triple-staining of corneal whole-mount in a WT C57BL/6 mouse with CD45, PDCA-1, and CD45R/B220 on day 3 following thermal cautery, indicating presence of $CD45^+$ PDCA-1^+ CD45R/$B220^+$ cells in the peripheral cornea (arrow).

(**P**) Quantification of $CD45^+$ PDCA-1^+ cells in naïve and inflamed C57BL/6 corneas (n=3-5/time point).

(**Q**) Representative confocal micrographs of PDCA-1^+ cells in sham-infected (upper panel) and HSV-1-infected (lower panel) corneas two days post intervention.

(**R**) Quantification of PDCA-1^+ cells in naïve, sham- and HSV-1-infected corneas (n=5/time point).

N: cell nucleus. G: Golgi apparatus. White asterisk: pDC thick process. Arrowhead: cell body. Scale bars: 100 μm (**I**, **O,** and **Q**), 50 μm (**C-H**, and **J-N**), 5 μm (**A**), and 2 μm (**B**). Bars denote SD. * $p < 0.05$

Supplementary Figure 3 (related to Figure 3-6)

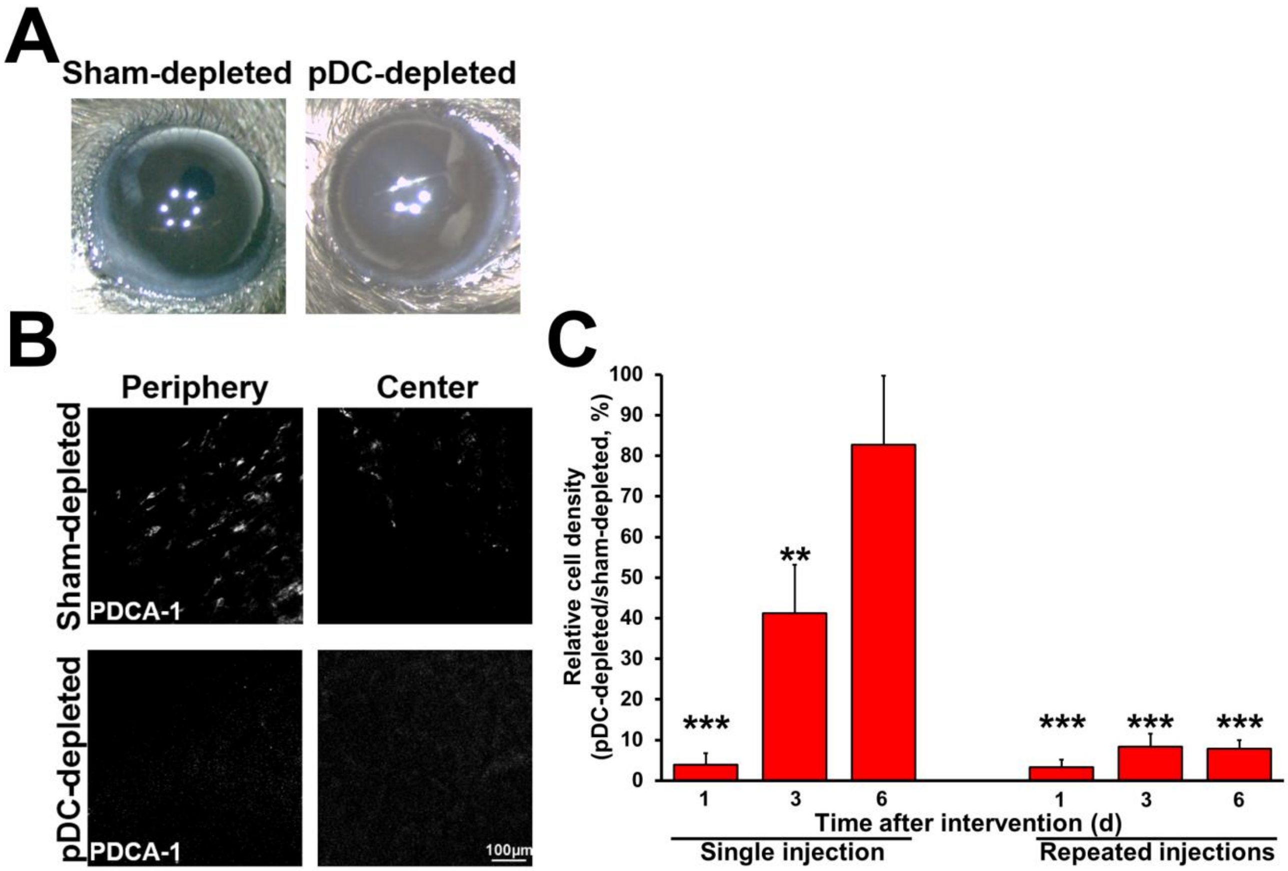

Figure S3. Local Depletion of Corneal pDCs (related to Figure 3-6).

(**A**) Representative clinical images of corneas of WT C57BL/6 and BDCA-2-DTR mice on day 1 following subconjunctival administration of 30 ng DT.

(**B-C**) Representative confocal micrographs of whole-mounted corneas stained with PDCA-1, 3 day following single injection in sham- (upper panel; **B**) and pDC-depleted corneas (lower panel; **B**). (**C**) Quantification of corneal PDCA-1^{+} cells (n=3/time point). Scale bar: 100 μm. Bars denote SD. ** p<0.01, *** p<0.001 (compared to sham-depleted controls).

Supplementary Figure 4 (related to Figure 3-6)

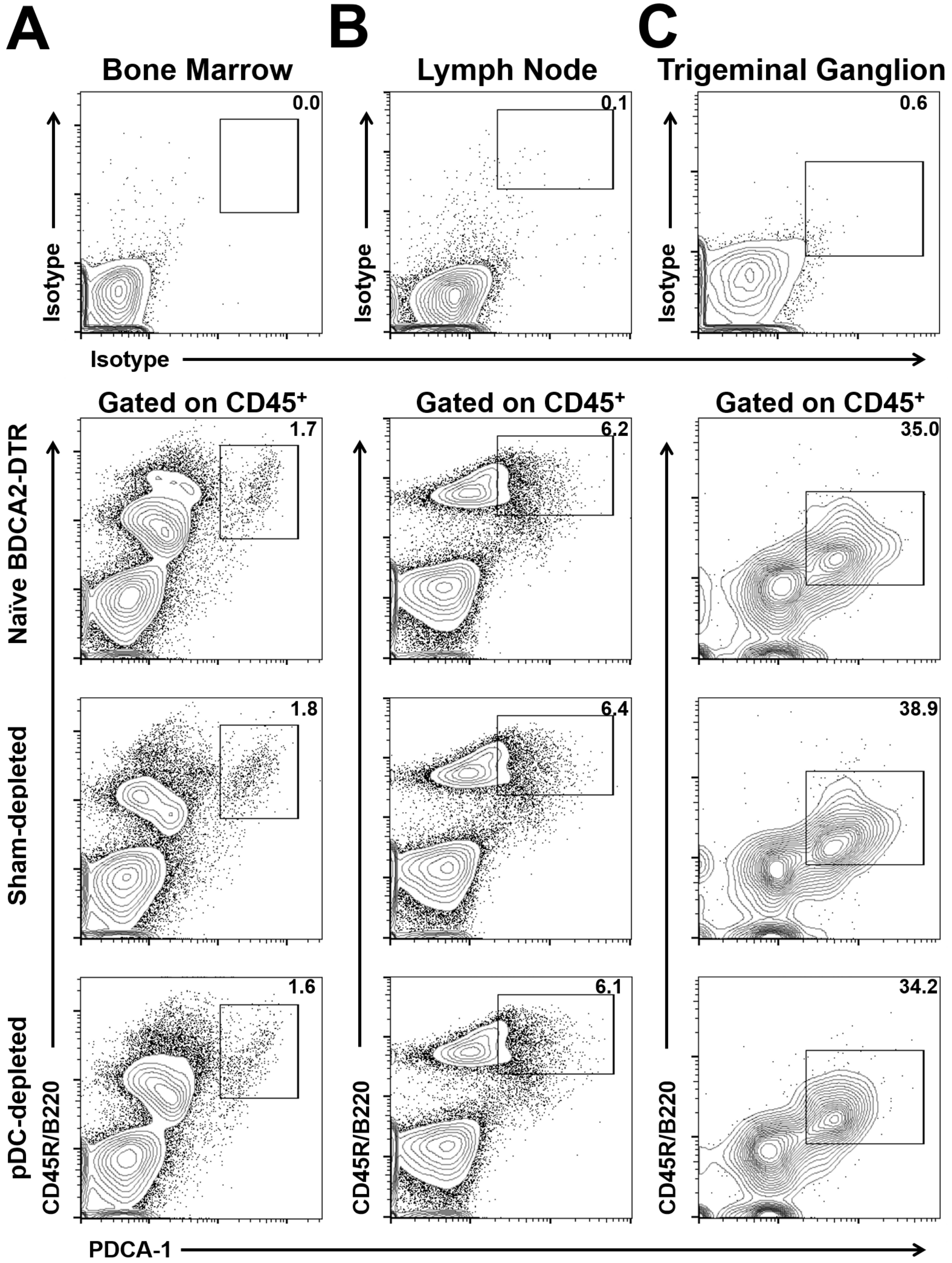

Figure S4. The Effect of Subconjuntival Injection of DT on the Density of pDCs in BMs, dLNs, and TGs of BDCA2-DTR and WT Mice (related to Figure 3-6)**.**

Representative flow cytometry plots in BM (**A**), mandibular dLN (**B**), and TG (**C**) in naïve BDCA-2-DTR mice as well as 48 h following subconjunctival injection of 30 ng DT in WT C57BL/6 (sham-depleted) and BDCA-2-DTR (pDC-depleted) mice after gating out debris, dead cells, and doublets, and subsequent gating on $CD45^+$ cells. The experiment was repeated three times.

Supplementary Figure 5 (related to Figure 5)

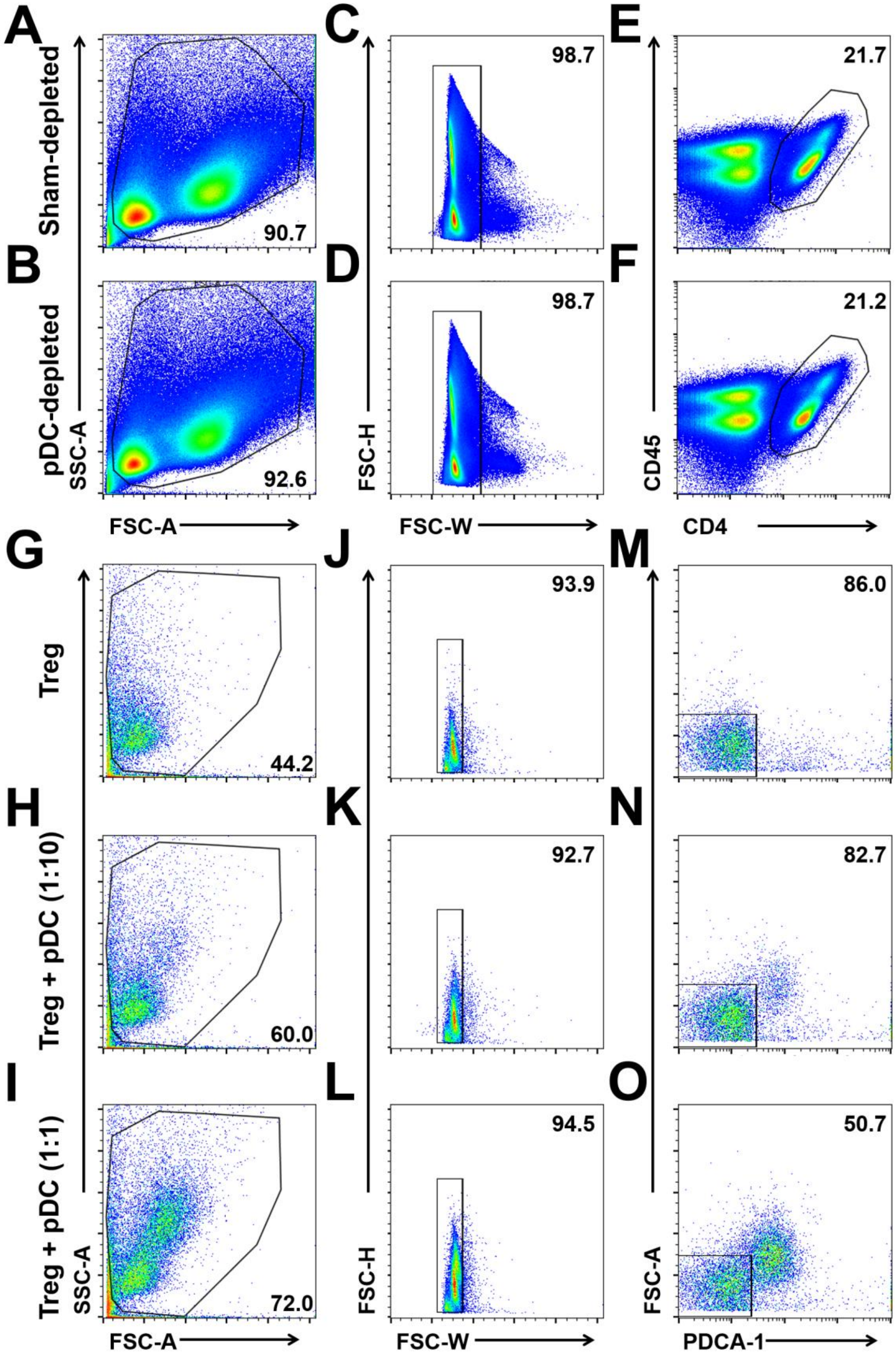

Figure S5. Flow Cytometry Gating Strategy for Studying pDCs and ex-Tregs (related to Figure 5)**.**

Flow cytometry dot-plots are shown for chimeric mice (**A-F**) and *in vitro* Treg and pDC co-culture studies (**G-O**). Gating strategy for removing dead cells and debris (**A**, **B,** and **G-I**), and doublets (**C, D** and **J-L**), followed by gating on $CD45^+$ $CD4^+$cells (**E** and **F**) or removing PDCA-1^+ pDCs (**M** and **O**) are presented. The experiment was repeated three times.

Supplementary Figure 6 (related to Figure 3-6)

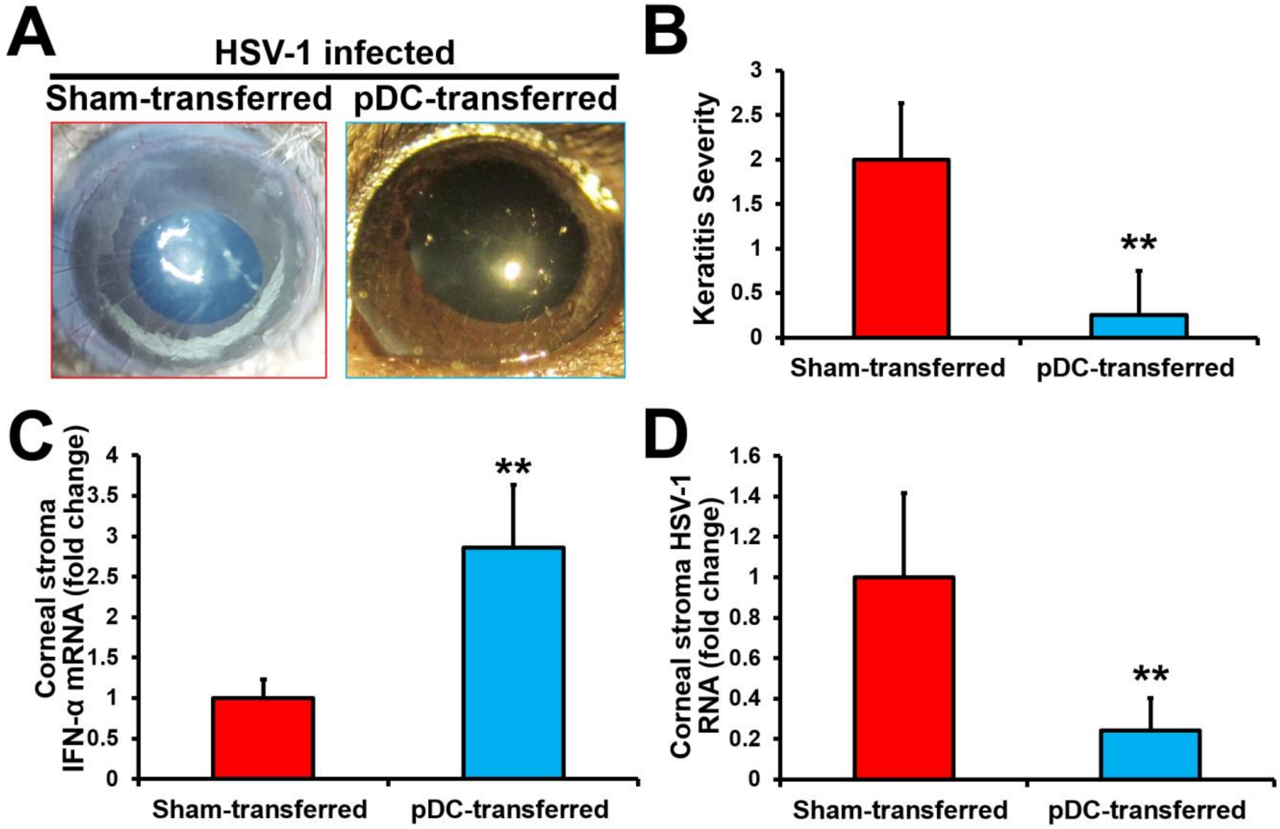

Figure S6. Local Adoptive Transfer of pDCs Diminishes Clinical Severity and Promotes Viral Clearance in Acute HSV-1 Keratitis (related to Figure 3-6)**.**

(**A**) Representative clinical images of corneas on day 5 following inoculation of HSV-1 in sham- and pDC-transferred mice.

(**B**) Quantification of clinical severity of HSV-1 keratitis in mice receiving pDCs compared to sham-transferred corneas (n=4-6/group).

(**C** and **D**) IFN-α mRNA (**C**) and HSV-1 gB RNA (**D**) in corneal stroma (n=4-6/group). Bars denote SD. ** $p<0.01$

Supplementary Figure 7 (related to Figure 1)

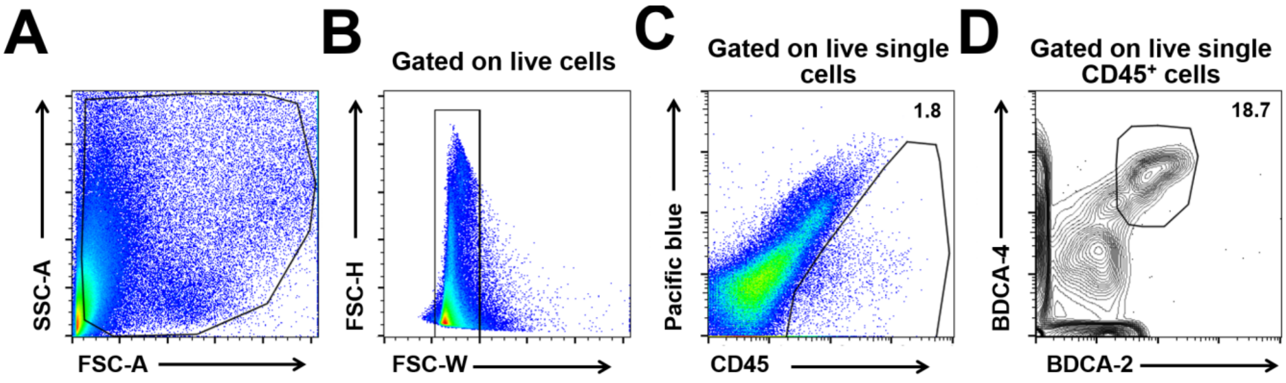

Figure S7. Human Corneas Host Resident pDCs (related to Figure 1)**.**

Representative flow cytometric dot plots on a digested human cornea demonstrating gating out debris and dead cells (**A**), selection of single cells (**B**), and gating on $CD45^+$ immune cells, followed by presenting $CD45^+$ BDCA-4^+ BDCA-2^+ resident pDCs (**D**). Plots are representative of three independent experiments.

Supplementary Table 1. Sequence of primers used in the study (relates to STAR Methods resource table)**.**

Transcript	Forward	Reverse
CD45R/B220	5’-AATGGCTCTTCAGAGACCACATA-3’	5’-AGTCAGGCTGTGGGGACA-3’
E2-2/Tcf4	5'-ACA ACG GAG CGA TGG GTA G-3'	5'-GCA GGA GAG AAT GGC TGC CT-3'
HSV-1 glycoprotein B	5’-AACGCGACGCACATCAAG-3’	5’-CTGGTACGCGATCAGAAAGC-3’
IFN-α1	5’-TCAATGACCTGCAAGGCTGTCTG-3’	5’-GGCATCTTCCTGGGTCAGGGGAAA-3’
TLR7	5'-AATCCACAGGCTCACCCATA-3'	5'-CAGGTACCAAGGGATGTCCT-3'
TLR9	5'-ACTGAGCACCCCTGCTTCTA -3'	5'AGATTAGTCAGCGGCAGGAA-3'
GAPDH	5’-CCCACTAACATCAAATGGGG-3’	5’-GATGATGACCCTTTTGGCTC-3’
